# Supplementary material for: Mortality in gastro-oesophageal reflux disease in a population-based nationwide cohort study of Swedish twins
Source: BMJ Open. 2020 Aug 6;10(8):e037456. doi: 10.1136/bmjopen-2020-037456 (PMC7412590; doi:10.1136/bmjopen-2020-037456)
Supplement: Author's manuscript [file bmjopen-2020-037456.draft_revisions.pdf]

BMJ Open is committed to open peer review. As part of this commitment we make the peer review history of every article we publish publicly available.

When an article is published we post the peer reviewers' comments and the authors' responses online. We also post the versions of the paper that were used during peer review. These are the versions that the peer review comments apply to.

The versions of the paper that follow are the versions that were submitted during the peer review process. They are not the versions of record or the final published versions. They should not be cited or distributed as the published version of this manuscript.

BMJ Open is an open access journal and the full, final, typeset and author-corrected version of record of the manuscript is available on our site with no access controls, subscription charges or pay-per-view fees (http://bmjopen.bmj.com).

If you have any questions on BMJ Open's open peer review process please email info.bmjopen@bmj.com

BMJ Open

# Mortality in gastro-oesophageal reflux disease in a nationwide Swedish twin study

| | |
|---|---|
| Journal: | *BMJ Open* |
| Manuscript ID | bmjopen-2020-037456 |
| Article Type: | Original research |
| Date Submitted by the Author: | 03-Feb-2020 |
| Complete List of Authors: | Ness-Jensen, Eivind; Karolinska Institute, Upper Gastrointestinal Research, Department of Molecular Medicine and Surgery; NTNU, HUNT Research Centre, Department of Public Health and Nursing<br>Santoni, Giola; Karolinska Institute, Upper Gastrointestinal Research, Department of Molecular Medicine and Surgery<br>Gottlieb-Vedi, Eivind; Karolinska Institute, Upper Gastrointestinal Research, Department of Molecular Medicine and Surgery<br>Lindam, Anna; Karolinska Institute, Upper Gastrointestinal Research, Department of Molecular Medicine and Surgery<br>Pedersen, Nancy; Karolinska Institute, Department of Medical Epidemiology and Biostatistics<br>Lagergren, Jesper; Karolinska Institute, Department of Molecular medicine and Surgery; King's College London, Division of Cancer Studies |
| Keywords: | EPIDEMIOLOGY, Oesophageal disease < GASTROENTEROLOGY, Gastrointestinal tumours < ONCOLOGY |
| | |

SCHOLARONE™
Manuscripts

*I, the Submitting Author has the right to grant and does grant on behalf of all authors of the Work (as defined in the below author licence), an exclusive licence and/or a non-exclusive licence for contributions from authors who are: i) UK Crown employees; ii) where BMJ has agreed a CC-BY licence shall apply, and/or iii) in accordance with the terms applicable for US Federal Government officers or employees acting as part of their official duties; on a worldwide, perpetual, irrevocable, royalty-free basis to BMJ Publishing Group Ltd ("BMJ") its licensees and where the relevant Journal is co-owned by BMJ to the co-owners of the Journal, to publish the Work in this journal and any other BMJ products and to exploit all rights, as set out in our licence.*

*The Submitting Author accepts and understands that any supply made under these terms is made by BMJ to the Submitting Author unless you are acting as an employee on behalf of your employer or a postgraduate student of an affiliated institution which is paying any applicable article publishing charge ("APC") for Open Access articles. Where the Submitting Author wishes to make the Work available on an Open Access basis (and intends to pay the relevant APC), the terms of reuse of such Open Access shall be governed by a Creative Commons licence – details of these licences and which Creative Commons licence will apply to this Work are set out in our licence referred to above.*

*Other than as permitted in any relevant BMJ Author's Self Archiving Policies, I confirm this Work has not been accepted for publication elsewhere, is not being considered for publication elsewhere and does not duplicate material already published. I confirm all authors consent to publication of this Work and authorise the granting of this licence.*

For peer review only

**Title: Mortality in gastro-oesophageal reflux disease in a nationwide Swedish twin study**

**Authors:** Eivind Ness-Jensen, MD, PhD[1,2,3], Giola Santoni, PhD[1], Eivind Gottlieb-Vedi, MD[1], Anna Lindam, PhD[1], Nancy L. Pedersen, PhD[4], Jesper Lagergren, MD, PhD[1,5]

**Affiliations and addresses:** [1]Upper Gastrointestinal Surgery, Department of Molecular medicine and Surgery, Karolinska Institutet, Karolinska University Hospital, S-171 76 Stockholm, Sweden;

[2]HUNT Research Centre, Department of Public Health and Nursing, NTNU, Norwegian University of Science and Technology, Forskningsvegen 2, N-7600 Levanger, Norway;

[3]Medical Department, Levanger Hospital, Nord-Trøndelag Hospital Trust, P.O. Box 333, N-7601 Levanger, Norway;

[4]Department of Medical Epidemiology and Biostatistics, Karolinska Institutet, S-171 76 Stockholm, Sweden;

[5]School of Cancer and Pharmaceutical Sciences, King's College London, London SE1 9RT, United Kingdom

**Corresponding author:** Eivind Ness-Jensen, eivind.ness-jensen@ki.se, Upper Gastrointestinal Surgery, Department of Molecular medicine and Surgery, Karolinska Institutet, Karolinska University Hospital, S-171 76 Stockholm, Sweden. Telephone: 0046-851776012

**Word count:** 2604



For peer review only - http://bmjopen.bmj.com/site/about/guidelines.xhtml

## Abstract

**Objectives:** The public health disorder gastro-oesophageal reflux disease (GORD) is linked with several comorbidities, including oesophageal adenocarcinoma, but whether life expectancy is reduced by GORD is uncertain. This study assessed all-cause and cancer-specific mortality in GORD after controlling for confounding by heredity and other factors.

**Design:** Population-based cohort study from 1998 through 2015.

**Setting:** Swedish nationwide study.

**Participants:** Twins (n=40,961) born in 1958 or earlier in Sweden.

**Exposure:** GORD symptoms reported in structured computer-assisted telephone interviews.

**Outcomes:** The primary outcome was all-cause mortality and the secondary outcome was cancer-specific mortality among twins with GORD and twins without GORD. Hazard ratios (HR) and 95% confidence intervals (CI) were analysed using parametric survival models, both in individual twin analyses and co-twin pair analyses, with adjustment for body mass index, smoking, education and comorbidity.

**Results:** Among 40,961 individual twins, 5,812 (14.2%) had GORD at baseline and 8,062 (19.7%) died during follow-up of up to 16 years. The risk of all-cause mortality (HR=1.00, 95% CI 0.94-1.07) and cancer-specific mortality (HR=0.99, 95% CI 0.89-1.10) were not increased in individual twins with GORD compared to individual twins without GORD. Similarly, there were no differences in mortality outcomes in within-pair analyses. The oesophageal adenocarcinoma-specific mortality rate was 0.45 (95% CI 0.32-0.66) per 1,000 person-years in individual twins with GORD and 0.22 (95% CI 0.18-0.27) per 1,000 person-years without GORD, rendering an adjusted HR of 2.01 (95% CI 1.35-2.98).


For peer review only

**Conclusions:** GORD did not increase all-cause or cancer-specific mortality when taking heredity and other confounders into account. The increased relative risk of mortality in oesophageal adenocarcinoma was low in absolute numbers.

**Keywords:** Survival; prognosis, heartburn; neoplasm; heredity; comorbidity

For peer review only

## Article Summary

### Strengths and limitations of this study

- The twin design which adjusts for heredity and shared familial confounders
- The prospective and nationwide population-based approach which counteracts recall and selection bias, as well as chance errors
- Valid and complete long-term follow-up using national registers
- Assessment of potential confounders
- No objective assessment of gastro-oesophageal reflux disease

For peer review only



For peer review only - http://bmjopen.bmj.com/site/about/guidelines.xhtml

## Introduction

Gastro-oesophageal reflux disease (GORD) is defined by troublesome heartburn and acid regurgitation occurring at least weekly or GORD-specific complications.[1] GORD affects between 10-30% of adults in the Western world and is one of the most common reasons for visits to gastroenterologists and general practitioners.[2, 3] Heredity, obesity, and tobacco smoking are the only established risk factors, while socioeconomic factors (mainly educational level) might also influence the risk of GORD.[4-7] Twin studies have shown that the heritability for GORD is 31-43%.[8, 9] Because GORD is associated with several conditions, e.g. cardiovascular disease, various gastrointestinal symptoms, anxiety, depression, sleep disorders,[10-13] reductions in health-related quality of life,[14, 15] and oesophageal and gastric cardia adenocarcinoma,[16] it has been hypothesised that GORD reduces life expectancy in general and increases mortality from cancer specifically. This is an important topic, not the least considering the high prevalence of GORD, and the consequences any influence on life expectancy would mean for healthcare and public health interventions. However, the research that has examined whether GORD increases the risk of mortality has been limited and provided conflicting results, some indicating a reduced survival and other not.[17-20] No previous study has taken influence of all risk factors for GORD into account as confounders, particularly not heredity or shared familial exposures.

The present study aimed to clarify whether GORD influences the mortality for all causes, cancer in general, and oesophageal adenocarcinoma in specific by conducting a large and comprehensive twin study, controlling for genetic and familial influences, together with other potential confounders.



For peer review only - http://bmjopen.bmj.com/site/about/guidelines.xhtml

## Methods

Study design

This population-based twin study was based on data from the Swedish Twin Registry, during the study period 1998 through 2015. This Swedish Twin registry incorporates comprehensive data retrieved directly from twins combined with data collected from Swedish national health registries. The personal identity number, which is assigned to each Swedish inhabitant, enabled exact linkage of participants' data between the data sources.[21] The study was approved by the Regional Ethical Review Board in Stockholm reference number 2010/582-31/1). All twins gave a broad informed consent for data collection and research when participating. The study protocol conforms to the ethical guidelines of the 1975 Declaration of Helsinki as reflected in a priori approval by the institution's human research committee.

Cohort

The study cohort was based on data from the Swedish Twin Registry, the largest and most comprehensive twin registry globally.[22, 23] It was established in the late 1950s and includes virtually all twins born in Sweden from 1886 onwards. During 1998-2002, the Screening Across the Lifespan Twin study (SALT) was performed with structured computer-assisted telephone interviews of twins born in 1958 or earlier and recorded in the Twin Registry, including assessment of GORD symptoms and risk factors for GORD.[22, 23] Data from the SALT interviews were used to define the study cohort and to assess information about GORD and the potential confounders heredity, body mass index (BMI), tobacco smoking, and education. Zygosity was assessed by a separate questionnaire sent to the twins. The twins were defined as monozygotic if both twins in a same-sexed pair reported they were "alike as



For peer review only - http://bmjopen.bmj.com/site/about/guidelines.xhtml

two peas in a pod" and as dizygotic if they reported to be "not more alike than siblings". This simple method has been shown to be 99% accurate in of determining zygosity compared to DNA-testing.[22] The Swedish Twin Registry is regularly updated with information from other nationwide Swedish registries, i.e. the Cause of Death Registry, Cancer Registry and Patient Registry, which are briefly presented below.

*The Swedish Cause of Death Registry* provided data on all-cause and cancer-specific mortality. This registry includes date of death and causes of death for all Swedish residents since 1961, regardless of whether they died in Sweden or abroad. The information about date of death and cause of death is 100% and 99% complete, respectively.[24, 25]

*The Swedish Cancer Registry* had information about the histological type of oesophageal cancer (adenocarcinoma). This registry started in 1958 and includes standardized records of all newly diagnosed malignancies in Sweden, including date of diagnosis, tumour site, and histological type. Histological type is registered in accordance with the World Health Organization's classification of histology (C24). The general completeness of the registry is 96% and it is 98% complete regarding recording of oesophageal adenocarcinoma, and for these patients, the histological verification is 100% complete.[26, 27]

*The Swedish Patient Registry* contained data on comorbidity. The registry contains date and International Classification of Diseases (ICD) versions 9 and 10 codes of diagnoses from all inpatient healthcare in Sweden from 1987 onwards and all specialist outpatient healthcare since 2001. This registry has a positive predictive value of any primary diagnosis close to 100%.[28] Diagnoses registered three years before and three years after the SALT interviews



For peer review only - http://bmjopen.bmj.com/site/about/guidelines.xhtml

were included in the assessment of comorbidity. This restriction in time was done to counteract misclassification of comorbidity due to different lengths of follow-up among the participating twins.

Exposure

The twins were defined as being exposed to GORD if they reported in the SALT interview to have: 1) heartburn at least weekly, 2) regurgitation at least weekly, or 3) retrosternal pain at least weekly combined with antacid relief.[1]

Outcomes

The main outcome was all-cause mortality, which included any deaths, regardless of cause. A secondary outcome was overall cancer-specific mortality, which included deaths related to any cancer (ICD-7 140-199 or ICD-10 C00-C97), excluding non-melanoma skin cancer (ICD-7 191 or ICD-10 C44). The other secondary outcome was oesophageal adenocarcinoma-specific mortality, defined as deaths related to oesophageal or gastroesophageal junctional adenocarcinoma (ICD-7 150 or 151.1 and C24 096 or ICD-10 C15 or C16.0 and C24 096).

Confounders

Data on BMI, tobacco smoking, and education were retrieved from the SALT interviews. BMI was calculated as the weight (kilograms) divided by the square height (meters). Smoking status included consumption of cigarettes, cigars, and pipe. The level of education was assessed by the highest reported completed education qualification. Data on comorbidity were collected from the Swedish Patient Registry. The last version of the Charlson comorbidity index was used to define and classify comborbidity.[29]



For peer review only - http://bmjopen.bmj.com/site/about/guidelines.xhtml

Statistical analyses

Mortality rates per 1,000 person-years were compared between individuals with and without GORD for all three mortality outcomes. Parametric survival models with Weibull distribution and sandwich estimator for the variance clustered by the twins' pair identity were used to calculate hazard ratios (HR) with 95% confidence intervals (CI). These models correct for within twin pair dependency and to avoid underestimation of the variance. The baseline hazard was modelled with a linear and a quadratic time term. Proportionality of the hazards was verified in all analyses. Time at risk was defined from the date of the SALT interview (1998-2002), i.e., when GORD was assessed, until the first date of death or the end of the study period (December 31, 2015).

The mortality among twins with GORD was compared with the mortality among twins without GORD in a stepwise series of analyses. First, external control analyses were performed using all individual twins, comparing individual twins with GORD to individual twins without GORD. Second, within-pair co-twin analyses of dizygotic twins discordant for GORD were performed. Third, within-pair co-twin analyses of monozygotic twins discordant for GORD were conducted. In the two latter analyses, only complete twin pairs were included. These three analysis steps were performed for each mortality outcome.

Stepwise adjustments for confounders were performed. First, a basic model adjusted for age (continuous) and sex. Second, the results were additionally adjusted for BMI (categorised into <25, 25-30, or >30), smoking (never, former, or current), and years of completed education (0-9.5 years, 9.5-12.5 years, or >12.5 years). Third, the results were further



For peer review only - http://bmjopen.bmj.com/site/about/guidelines.xhtml

adjusted for comorbidity (Charlson comorbidity index score 0, 1, or ≥2),[29] which was done to assess whether comorbidity could explain any association between GORD and mortality.

In order to examine effect modification, analyses were stratified by age (≤60 or >60 years) and sex (except for the monozygotic twin analyses). In the monozygotic twin analysis of men aged 40-60 years, the HRs were estimated with exponential distribution and sandwich estimator for the variance, clustered by the twins' pair identity in order for the model to converge. This result should be similar to the model with Weibull distribution, which did not converge in this analysis.

A senior biostatistician (GS) conducted the data management and statistical analysis following a pre-defined study protocol. The statistical analyses were performed using Stata MP version 15, StataCorp LP, College Station, TX, USA.

For peer review only



For peer review only - http://bmjopen.bmj.com/site/about/guidelines.xhtml

## Results

Participants

Among 43,350 individual twins who participated in SALT, 40,961 (95.5%) answered the questions relevant for the present study and were thus included in the final analysis. The figure shows a flowchart describing the study cohort. Among the participating twins, 8,062 (19.7%) died during follow-up of up to 16 years, including 2,845 (6.9%) from any cancer and 127 (0.3%) from oesophageal adenocarcinoma. Characteristics of the included twins with and without GORD are shown in Table 1. The median age was 56 years in both groups. In all, 14.2% had GORD and GORD was similarly common in both sexes and in both dizygotic and monozygotic twins. Compared to twins without GORD, the twins with GORD were more often overweight or obese, tobacco smokers, less educated, and diagnosed with comorbidities (Table 1). The study included 2,501 dizygotic twin pairs discordant for GORD and 749 monozygotic twin pairs discordant for GORD.

Mortality from any cause

The all-cause mortality rate of all individual twins was 16.2 (95% CI 15.3-17.2) per 1,000 person-years in twins with GORD and also 16.2 (95% CI 15.8-16.7) per 1,000 person-years in twins without GORD (Table 2). In dizygotic twin pairs discordant for GORD, the all-cause mortality rates were 13.3 (95% CI 12.1-14.7) per 1,000 person-years in twins with GORD and 13.3 (95% CI 12.2-14.7) per 1,000 person-years for their co-twins without GORD. In monozygotic twin pairs discordant for GORD, the all-cause mortality rates were 12.0 (95% CI 10.0-14.4) per 1,000 person-years in twins with GORD and 12.2 (95% CI 10.2-14.6) per 1,000 person-years in their co-twins without GORD.



For peer review only - http://bmjopen.bmj.com/site/about/guidelines.xhtml

The fully adjusted HR of all-cause mortality was 1.00 (95% CI 0.94-1.07) comparing all individual twins with GORD to individual twins without GORD (Table 3). In the dizygotic twin analysis, the corresponding HR was 0.99 (95% CI 0.87-1.14). In the monozygotic twin analysis, the adjusted HR was 1.11 (95% CI 0.87-1.40). The analyses stratified by sex and age showed similar HRs without any association between GORD and all-cause mortality (Table 4).

Mortality from any cancer

The overall cancer-specific mortality rate of all individual twins was 5.8 (95% CI 5.3-6.4) per 1,000 person-years in those with GORD and 5.7 (95% CI 5.5-6.0) per 1,000 person-years in those without GORD (Table 2). The dizygotic twin analysis also showed similar cancer-specific mortality rates in twins with GORD (4.9 [95% CI 4.2-5.7] per 1,000 person-years) and their co-twin without (4.9 8 [95% CI 4.2-5.7] per 1,000 person-years). In the monozygotic twin analysis, the corresponding rates were 5.2 (95% CI 3.9-6.9) per 1,000 person-years in twins with GORD and 4.6 (95% CI 3.5-6.3) per 1,000 person-years in their co-twins with no GORD.

The fully adjusted HR of overall cancer-specific mortality was 0.99 (95% CI 0.89-1.10) comparing all individual twins with GORD to individual twins without GORD (Table 3). The corresponding HRs in dizygotic twins and monozygotic twins were 0.99 (95% CI 0.78-1.24) and 1.28 (95% CI 0.87-1.87), respectively. The analyses stratified by sex and age showed similar HRs and no association between GORD and overall cancer-specific mortality (Table 4).



For peer review only - http://bmjopen.bmj.com/site/about/guidelines.xhtml

For peer review only

Mortality from oesophageal adenocarcinoma

The oesophageal adenocarcinoma-specific mortality rate was 0.45 (95% CI 0.32-0.66) per 1,000 person-years in all individual twins with GORD, compared to 0.22 (95% CI 0.18-0.27) per 1,000 person-years in twins without GORD (Table 2). In dizygotic twins, this rate was 0.39 (95% CI 0.23-0.74) per 1,000 person-years in the twins with GORD and 0.26 (95% CI 0.13-0.58) per 1,000 person-years in the twins without GORD. The mortality rate was 0.32 (95% CI 0.10-1.58) per 1,000 person-years in the monozygotic twins with GORD, while there was no oesophageal adenocarcinoma-specific mortality in the monozygotic twins without GORD.

The fully adjusted HR was 2.01 (95% CI 1.35-2.98) for oesophageal adenocarcinoma-specific mortality comparing all individual twins with GORD to those without GORD (Table 3). In dizygotic twins, the corresponding HR was 1.44 (95% CI 0.60-3.45), while the statistical power was insufficient for monozygotic twin analysis. The HR was 3.71 (95% CI 1.90-7.28) in men aged 40-60 years, and 1.60 (95% CI 0.77-3.32) in men aged >60 years (Table 4). The stratified dizygotic twin analyses had low statistical power, but the fully adjusted HR for oesophageal adenocarcinoma-specific mortality was 2.07 (95% CI 0.53-8.08) among men aged 40-60 years and 0.82 (95% CI 0.15-4.61) among men aged >60 years.

For peer review only

## Discussion

This large-scale twin study found no increased all-cause or cancer-specific mortality in twins with GORD compared to twins without GORD. The risk of mortality in oesophageal adenocarcinoma was higher in twins with GORD than in twins without GORD, but the absolute risk was still low.

Among methodological strengths is the twin design, which enabled the first study on the topic with adjustment for heredity and shared familial confounders. The prospective and nationwide population-based approach counteracted recall and selection bias, as well as chance errors. The high-quality and complete data reduced misclassification and enabled long and complete follow-up of all participants. The assessment of mortality was valid and complete. The definition of GORD was the evidence-based Montreal consensus, which remains the definition of choice for research purposes.[1] The prevalence of GORD in this study coincides well with the prevalence reported in similar Western populations,[2] indicating validity of the definition of GORD. The assessment of potential confounders through the structured SALT interviews (BMI, tobacco smoking, and education) and the Patient Registry (comorbidity) allowed for adjustment of all risk factors for GORD and mortality, i.e. all plausible confounders. Yet, a weakness is residual confounding, which cannot be entirely ruled out in this observational study. The rate of missing values for the variables included in the study was low, and all analyses were complete case analyses. The large sample size allowed for age and sex stratified analyses to assess effect modification with age and sex, but the dizygotic and monozygotic co-twin analyses had limited statistical power, although the results generally supported the overall findings.



For peer review only - http://bmjopen.bmj.com/site/about/guidelines.xhtml

The results of the present study showing no increased all-cause mortality in individuals with GORD corroborates the findings of our recent cohort study from Norway,[20] a cohort study from the United States,[18] and a cohort study from Iran.[19] However, three cohort studies from the United Kingdom showed a 1.16- to 1.6-fold increase in mortality in people with GORD compared with the background population, the majority of deaths being due to cardiac disease.[17] The increased mortality found in some studies could be due to prevalent cancers provoking GORD symptoms. No earlier study has heredity as a confounder, although heredity is a strong risk factor for GORD.[8]

GORD is common in Western populations, with 10-30% prevalence in adults [2, 3]. The present study implies that individuals with GORD do not need to worry about any increased risk of dying. The increased risk of death from oesophageal adenocarcinoma should not be overemphasized because the absolute risk is still low even in the presence of GORD. However, if the incidence of oesophageal adenocarcinoma continues to increase strongly without any improvements in the survival, the influence of mortality from this tumour could increase.

In conclusion, this nationwide Swedish population-based cohort study in twins with long and complete follow-up and adjustment for confounders indicates that GORD does not increase the risk of all-cause or cancer-specific mortality. Despite the increased relative risk of mortality from oesophageal adenocarcinoma in individuals with GORD, the absolute risk is still low.



For peer review only - http://bmjopen.bmj.com/site/about/guidelines.xhtml

**Acknowledgments**

We are grateful to the Swedish Twin Registry for access to data. This Registry is managed by Karolinska Institutet and receives funding from the Swedish Research Council (grant 2017-00641).

**Disclosure of interest**

The authors report no conflict of interest.

**Funding**

This work was supported by the Swedish Research Council (SIMSAM) under grant number 2017-00641; Swedish Society of Medicine; and the United European Gastroenterology (UEG Research Prize to JL). The funders did not have any role in the study design, in the collection, analysis, or interpretation of data.

**Data availability statement**

The data set used in this paper is available through application to The Swedish Twin Registry (https://ki.se/en/research/swedish-twin-registry-for-researchers)

**Author statement**

(i) Guarantor of the article: Eivind Ness-Jensen; (ii) Specific author contributions: **ENJ:** study concept and design; acquisition of data; analysis and interpretation of data; drafting of the manuscript; critical revision of the manuscript for important intellectual content; obtained funding. **GS:** study concept and design; analysis and interpretation of data; critical revision of the manuscript for important intellectual content; statistical analysis. **EGV:** study concept



For peer review only - http://bmjopen.bmj.com/site/about/guidelines.xhtml

and design; acquisition of data; analysis and interpretation of data; critical revision of the manuscript for important intellectual content. **AL:** study concept and design; acquisition of data; analysis and interpretation of data; critical revision of the manuscript for important intellectual content. **NP:** study concept and design; acquisition of data; analysis and interpretation of data; critical revision of the manuscript for important intellectual content. **JL:** study concept and design; analysis and interpretation of data; critical revision of the manuscript for important intellectual content; obtained funding; (iii) All authors approved the final version of the manuscript.

**Patient and Public Involvement statement**

Patients or the public were not involved in the design, or conduct, or reporting, or dissemination plans of our research.

For peer review only



For peer review only - http://bmjopen.bmj.com/site/about/guidelines.xhtml

## References

1. Vakil N, van Zanten SV, Kahrilas P, et al. The Montreal definition and classification of gastroesophageal reflux disease: a global evidence-based consensus. Am J Gastroenterol 2006;101:1900-20; quiz 1943.
2. El-Serag HB, Sweet S, Winchester CC, et al. Update on the epidemiology of gastro-oesophageal reflux disease: a systematic review. Gut 2014;63:871-80.
3. Ness-Jensen E, Lindam A, Lagergren J, et al. Changes in prevalence, incidence and spontaneous loss of gastro-oesophageal reflux symptoms: a prospective population-based cohort study, the HUNT study. Gut 2012;61:1390-7.
4. Dent J, El-Serag HB, Wallander MA, et al. Epidemiology of gastro-oesophageal reflux disease: a systematic review. Gut 2005;54:710-7.
5. Nilsson M, Johnsen R, Ye W, et al. Lifestyle related risk factors in the aetiology of gastro-oesophageal reflux. Gut 2004;53:1730-5.
6. Jansson C, Nordenstedt H, Johansson S, et al. Relation between gastroesophageal reflux symptoms and socioeconomic factors: a population-based study (the HUNT Study). Clin Gastroenterol Hepatol 2007;5:1029-34.
7. Hallan A, Bomme M, Hveem K, et al. Risk factors on the development of new-onset gastroesophageal reflux symptoms. A population-based prospective cohort study: the HUNT study. Am J Gastroenterol 2015;110:393-400; quiz 401.
8. Cameron AJ, Lagergren J, Henriksson C, et al. Gastroesophageal reflux disease in monozygotic and dizygotic twins. Gastroenterology 2002;122:55-9.
9. Mohammed I, Cherkas LF, Riley SA, et al. Genetic influences in gastro-oesophageal reflux disease: a twin study. Gut 2003;52:1085-9.
10. Jansson C, Nordenstedt H, Wallander MA, et al. Severe gastro-oesophageal reflux symptoms in relation to anxiety, depression and coping in a population-based study. Aliment Pharmacol Ther 2007;26:683-91.
11. Jansson C, Nordenstedt H, Wallander MA, et al. Severe symptoms of gastro-oesophageal reflux disease are associated with cardiovascular disease and other gastrointestinal symptoms, but not diabetes: a population-based study. Aliment Pharmacol Ther 2008;27:58-65.
12. Lindam A, Jansson C, Nordenstedt H, et al. A population-based study of gastroesophageal reflux disease and sleep problems in elderly twins. PLoS One 2012;7:e48602.
13. Lindam A, Ness-Jensen E, Jansson C, et al. Gastroesophageal Reflux and Sleep Disturbances: A Bidirectional Association in a Population-Based Cohort Study, The HUNT Study. Sleep 2016;39:1421-7.
14. Wiklund I, Carlsson J, Vakil N. Gastroesophageal reflux symptoms and well-being in a random sample of the general population of a Swedish community. Am J Gastroenterol 2006;101:18-28.
15. Ronkainen J, Aro P, Storskrubb T, et al. Gastro-oesophageal reflux symptoms and health-related quality of life in the adult general population--the Kalixanda study. Aliment Pharmacol Ther 2006;23:1725-33.
16. Lagergren J, Bergstrom R, Lindgren A, et al. Symptomatic gastroesophageal reflux as a risk factor for esophageal adenocarcinoma. N Engl J Med 1999;340:825-31.
17. Becher A, El-Serag HB. Mortality associated with gastroesophageal reflux disease and its non-malignant complications: a systematic review. Scand J Gastroenterol 2008;43:645-53.
18. Talley NJ, Locke GR, 3rd, McNally M, et al. Impact of gastroesophageal reflux on survival in the community. Am J Gastroenterol 2008;103:12-9.
19. Islami F, Pourshams A, Nasseri-Moghaddam S, et al. Gastroesophageal Reflux Disease and overall and Cause-specific Mortality: A Prospective Study of 50000 Individuals. Middle East J Dig Dis 2014;6:65-80.



For peer review only - http://bmjopen.bmj.com/site/about/guidelines.xhtml

20. Ness-Jensen E, Gottlieb-Vedi E, Wahlin K, et al. All-cause and cancer-specific mortality in GORD in a population-based cohort study (the HUNT study). Gut 2018;67:209-215.
21. Ludvigsson JF, Otterblad-Olausson P, Pettersson BU, et al. The Swedish personal identity number: possibilities and pitfalls in healthcare and medical research. Eur J Epidemiol 2009;24:659-67.
22. Lichtenstein P, De Faire U, Floderus B, et al. The Swedish Twin Registry: a unique resource for clinical, epidemiological and genetic studies. J Intern Med 2002;252:184-205.
23. Lichtenstein P, Sullivan PF, Cnattingius S, et al. The Swedish Twin Registry in the third millennium: an update. Twin Res Hum Genet 2006;9:875-82.
24. Johansson LA, Westerling R. Comparing Swedish hospital discharge records with death certificates: implications for mortality statistics. Int J Epidemiol 2000;29:495-502.
25. Brooke HL, Talback M, Hornblad J, et al. The Swedish cause of death register. Eur J Epidemiol 2017;32:765-773.
26. Barlow L, Westergren K, Holmberg L, et al. The completeness of the Swedish Cancer Register: a sample survey for year 1998. Acta Oncol 2009;48:27-33.
27. Lindblad M, Ye W, Lindgren A, et al. Disparities in the classification of esophageal and cardia adenocarcinomas and their influence on reported incidence rates. Ann Surg 2006;243:479-85.
28. Ludvigsson JF, Andersson E, Ekbom A, et al. External review and validation of the Swedish national inpatient register. BMC Public Health 2011;11:450.
29. Armitage JN, van der Meulen JH, Royal College of Surgeons Co-morbidity Consensus G. Identifying co-morbidity in surgical patients using administrative data with the Royal College of Surgeons Charlson Score. Br J Surg 2010;97:772-81.

For peer review only



For peer review only - http://bmjopen.bmj.com/site/about/guidelines.xhtml

**Table 1.** Baseline characteristics of twins with and without gastro-oesophageal reflux disease (GORD)

| | **GORD** | | **No GOERD** | |
|---|---|---|---|---|
| | Number (%) | | Number (%) | |
| Total | 5,812 | (14.2) | 35,149 | (85.8) |
| Age, years* | 56 | (41-95) | 56 | (41-99) |
| Sex | | | | |
| Men | 2,673 | (46.0) | 16,683 | (47.5) |
| Women | 3,139 | (54.0) | 18,466 | (52.5) |
| Zygosity | | | | |
| Monozygotic | 1,444 | (24-8) | 8,860 | (25.2) |
| Dizygotic | 4,368 | (75.2) | 26,289 | (75.8) |
| BMI | | | | |
| <25 | 2,568 | (44.2) | 19,577 | (55.7) |
| 25-30 | 2,535 | (43.6) | 12,909 | (36.7) |
| >30 | 709 | (12.2) | 2,663 | (7.6) |
| Tobacco smoking-status | | | | |
| Never | 1,236 | (21.3) | 8,985 | (25.6) |
| Former | 3,330 | (57.3) | 19,104 | (54.4) |
| Current | 1,246 | (21.4) | 7,060 | (20.1) |
| Education, years | | | | |
| 0-9.5 | 3,019 | (51.9) | 16,405 | (46.7) |
| 9.5-12.5 | 1,625 | (28.0) | 9,895 | (28.2) |
| >12.5 | 1,168 | (20.1) | 1,168 | (25.2) |
| Charlson co-morbidity index | | | | |
| 0 | 5,190 | (89.3) | 31,820 | (90.5) |
| 1 | 524 | (9.0) | 2,883 | (8.2) |
| >1 | 98 | (1.7) | 446 | (1.3) |

*Median (range

For peer review only

**Table 2.** Number of deaths and mortality rates for all-cause, cancer-specific, and oesophageal adenocarcinoma (OAC)-specific mortality in twins with and without gastro-oesophageal reflux disease (GORD)

| | **Alive (number)** | | **Deaths (number)** | | **Mortality rates per 1,000 person-years (95% CI)** | |
|---|---|---|---|---|---|---|
| **Outcome** | **GORD** | **No GORD** | **GORD** | **No GORD** | **GORD** | **No GORD** |
| All-cause mortality | | | | | | |
| All twins | 6,922 | 28,227 | 1,140 | 4,672 | 16.2 (15.3-17.2) | 16.2 (15.8-16.7) |
| Dizygotic[a] | 2,091 | 2,090 | 410 | 411 | 13.3 (12.1-14.7) | 13.3 (12.2-14.7) |
| Monozygotic[a] | 638 | 636 | 111 | 113 | 12.0 (10.0-14.4) | 12.2 (10.2-14.6) |
| Overall cancer-specific mortality | | | | | | |
| All twins | 5,404 | 32,707 | 408 | 2437 | 5.8 (5.3-6.4) | 5.7 (5.5-6.0) |
| Dizygotic[a] | 2,351 | 2,351 | 150 | 150 | 4.9 (4.2-5.7) | 4.9 (4.2-5.7) |
| Monozygotic[a] | 701 | 706 | 48 | 43 | 5.2 (3.9-6.9) | 4.6 (3.5-6.3) |
| OAC-specific mortality | | | | | | |
| All twins | 5,780 | 35,049 | 32 | 95 | 0.45 (0.32-0.66) | 0.22 (0.18-0.27) |
| Dizygotic[a] | 2,489 | 2,493 | 12 | 8 | 0.39 (0.23-0.74) | 0.26 (0.13-0.58) |
| Monozygotic[a] | 746 | 749 | 3 | 0 | 0.32 (0.10-1.58) | - |

[a]Discordant for gastro-oesophageal reflux disease



For peer review only - http://bmjopen.bmj.com/site/about/guidelines.xhtml

**Table 3.** Hazard ratio (HR) with 95% confidence interval (CI) for all-cause, cancer-specific, and oesophageal adenocarcinoma (OAC)-specific-specific mortality in twins with and without gastro-oesophageal reflux disease

| | Model 1[a] | | | Model 2[b] | | | Model 3[c] | | |
|---|---|---|---|---|---|---|---|---|---|
| **Outcome** | **HR** | **(95% CI)** | | **HR** | **(95% CI)** | | **HR** | **(95% CI)** | |
| All-cause mortality | | | | | | | | | |
| All twins | 1.03 | (0.97 | 1.10) | 1.03 | (0.96 | 1.09) | 1.00 | (0.94 | 1.07) |
| Dizygotic[d] | 0.99 | (0.87 | 1.13) | 1.04 | (0.91 | 1.18) | 0.99 | (0.87 | 1.14) |
| Monozygotic[d] | 0.99 | (0.79 | 1.24) | 1.05 | (0.84 | 1.32) | 1.11 | (0.87 | 1.40) |
| Overall cancer-specific mortality | | | | | | | | | |
| All twins | 1.04 | (0.93 | 1.15) | 1.02 | (0.92 | 1.14) | 0.99 | (0.89 | 1.10) |
| Dizygotic[d] | 1.00 | (0.80 | 1.25) | 1.04 | (0.83 | 1.30) | 0.99 | (0.78 | 1.24) |
| Monozygotic[d] | 1.13 | (0.78 | 1.62) | 1.21 | (0.84 | 1.75) | 1.28 | (0.87 | 1.87) |
| OAC-specific mortality | | | | | | | | | |
| All twins | 2.09 | (1.40 | 3.13) | 2.11 | (1.41 | 3.15) | 2.01 | (1.35 | 2.98) |
| Dizygotic[d] | 1.50 | (0.61 | 3.68) | 1.62 | (0.70 | 3.78) | 1.44 | (0.60 | 3.45) |
| Monozygotic[d] | - | - | - | - | - | - | - | - | - |

[a]Adjusted for age and sex

[b]Adjusted for age, sex, BMI, tobacco smoking status, and education

[c]Adjusted for age, sex, BMI, tobacco smoking status, education, and Charlson comorbidity index

[d]Discordant for gastro-oesophageal reflux disease

For peer review only

**Table 4.** Hazard ratio[a] (HR) with 95% confidence interval (CI) for all-cause, cancer-specific, and oesophageal adenocarcinoma (OAC)-specific mortality in twins with and without gastro-oesophageal reflux disease (GORD)

| | Age 40-60 years | | | | | Age >60 years | | | | |
|---|---|---|---|---|---|---|---|---|---|---|
| | Number of deaths | | | | | Number of deaths | | | | |
| **Outcome** | **GORD** | **No GORD** | **HR** | **(95% CI)** | | **GORD** | **No GORD** | **HR** | **(95% CI)** | |
| **Men** | | | | | | | | | | |
| All-cause mortality | | | | | | | | | | |
| All twins | 140 | 766 | 0.97 | (0.80 | 1.17) | 437 | 2,885 | 0.96 | (0.87 | 1.06) |
| Dizygotic twins[a] | 58 | 54 | 0.96 | (0.66 | 1.39) | 152 | 164 | 0.91 | (0.73 | 1.13) |
| Monozygotic twins[a] | 16 | 16 | 1.09 | (0.57 | 2.15) | 38 | 32 | 1.40 | (0.92 | 2.13) |
| Overall cancer-specific mortality | | | | | | | | | | |
| All twins | 61 | 327 | 0.97 | (0.73 | 1.29) | 151 | 971 | 0.97 | (0.81 | 1.15) |
| Dizygotic twins[a] | 27 | 24 | 1.06 | (0.60 | 1.87) | 51 | 57 | 0.80 | (0.54 | 1.18) |
| Monozygotic twins[a] | 3 | 5 | 0.65 | (0.12 | 3.38)[e] | 20 | 13 | 1.80 | (0.96 | 3.38) |
| OAC-specific mortality | | | | | | | | | | |
| All twins | 14 | 22 | 3.71 | (1.90 | 7.28) | 9 | 36 | 1.60 | (0.77 | 3.32) |
| Dizygotic twins[a] | 6 | 1 | 2.07 | (0.53 | 8.08) | 2 | 3 | 0.82 | (0.15 | 4.61) |
| Monozygotic twins[a] | 1 | 0 | - | - | - | 2 | 0 | - | - | - |
| **Women** | | | | | | | | | | |
| All-cause mortality | | | | | | | | | | |
| All twins | 133 | 676 | 1.03 | (0.85 | 1.26) | 430 | 2,595 | 1.00 | (0.90 | 1.11) |
| Dizygotic twins[a] | 52 | 56 | 1.03 | (0.70 | 1.51) | 148 | 137 | 1.10 | (0.87 | 1.40) |
| Monozygotic twins[a] | 15 | 20 | 0.75 | (0.34 | 1.67) | 42 | 45 | 1.11 | (0.78 | 1.57) |
| Overall cancer-specific mortality | | | | | | | | | | |
| All twins | 65 | 427 | 0.80 | (0.61 | 1.05) | 131 | 712 | 1.10 | (0.91 | 1.33) |
| Dizygotic twins[a] | 23 | 28 | 0.93 | (0.52 | 1.65) | 49 | 41 | 1.30 | (0.84 | 2.03) |
| Monozygotic twins[a] | 11 | 11 | 1.07 | (0.41 | 2.77) | 14 | 14 | 1.06 | (0.52 | 2.15) |
| OAC-specific mortality | | | | | | | | | | |
| All twins | 1 | 9 | 0.51 | (0.06 | 4.09) | 8 | 28 | 1.81 | (0.83 | 3.94) |
| Dizygotic twins[a] | 0 | 0 | - | - | - | 3 | 2 | 1.39 | (0.30 | 6.43) |
| Monozygotic twins[a] | 0 | 0 | - | - | - | 0 | 0 | - | - | - |

[a]Discordant for gastro-oesophageal reflux disease



For peer review only - http://bmjopen.bmj.com/site/about/guidelines.xhtml

**Figure.** Study population, sample, and vital status in twins with and without gastro-oesophageal reflux disease (GORD)

*SALT: Screening Across the Lifespan Twin cohort

§OAC: Oesophageal adenocarcinom

For peer review only

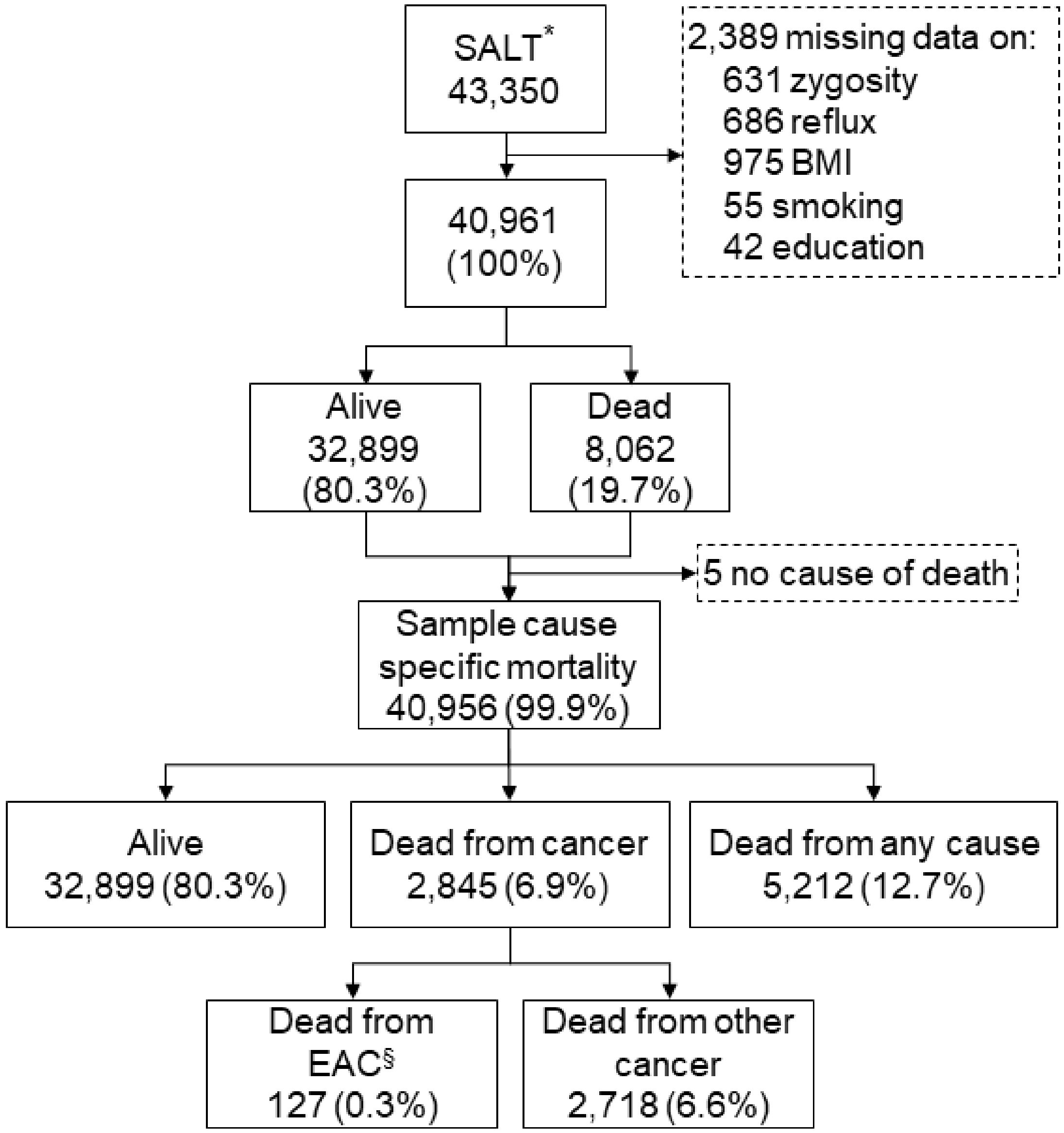
SALT*
43,350
2,389 missing data on:
631 zygosity
686 reflux
975 BMI
55 smoking
42 education
40,961
(100%)
Alive
32,899
(80.3%)
Dead
8,062
(19.7%)
5 no cause of death
Sample cause specific mortality
40,956 (99.9%)
Alive
32,899 (80.3%)
Dead from cancer
2,845 (6.9%)
Dead from any cause
5,212 (12.7%)
Dead from EAC§
127 (0.3%)
Dead from other cancer
2,718 (6.6%)

STROBE Statement—Checklist of items that should be included in reports of ***cohort studies***

| | Item No | Recommendation |
|---|---|---|
| **Title and abstract** | 1 | (*a*) Indicate the study's design with a commonly used term in the title or the abstract<br>**Page 1** |
| | | (*b*) Provide in the abstract an informative and balanced summary of what was done and what was found<br>**Page 2** |
| **Introduction** | | |
| Background/rationale | 2 | Explain the scientific background and rationale for the investigation being reported<br>**Page 5** |
| Objectives | 3 | State specific objectives, including any prespecified hypotheses<br>**Page 5** |
| **Methods** | | |
| Study design | 4 | Present key elements of study design early in the paper<br>**Page 6** |
| Setting | 5 | Describe the setting, locations, and relevant dates, including periods of recruitment, exposure, follow-up, and data collection<br>**Page 6** |
| Participants | 6 | (*a*) Give the eligibility criteria, and the sources and methods of selection of participants. Describe methods of follow-up<br>**Pages 6 to 7** |
| | | (*b*) For matched studies, give matching criteria and number of exposed and unexposed<br>**NA** |
| Variables | 7 | Clearly define all outcomes, exposures, predictors, potential confounders, and effect modifiers. Give diagnostic criteria, if applicable<br>**Pages 6 to 8** |
| Data sources/ measurement | 8* | For each variable of interest, give sources of data and details of methods of assessment (measurement). Describe comparability of assessment methods if there is more than one group<br>**Pages 6 to 8** |
| Bias | 9 | Describe any efforts to address potential sources of bias<br>**Pages 9 to 10** |
| Study size | 10 | Explain how the study size was arrived at<br>**Page 11 and Figure** |
| Quantitative variables | 11 | Explain how quantitative variables were handled in the analyses. If applicable, describe which groupings were chosen and why<br>**Page 9** |
| Statistical methods | 12 | (*a*) Describe all statistical methods, including those used to control for confounding<br>**Pages 9 to 10** |
| | | (*b*) Describe any methods used to examine subgroups and interactions<br>**Pages 9 to 10** |
| | | (*c*) Explain how missing data were addressed<br>**Page 14 and Figure** |
| | | (*d*) If applicable, explain how loss to follow-up was addressed<br>**NA** |
| | | (*e*) Describe any sensitivity analyses |

For peer review only

| | | |
|---|---|---|
| | | **NA** |
| **Results** | | |
| Participants | 13* | (a) Report numbers of individuals at each stage of study—eg numbers potentially eligible, examined for eligibility, confirmed eligible, included in the study, completing follow-up, and analysed **Page 11 and Figure** |
| | | (b) Give reasons for non-participation at each stage **Page 11 and Figure** |
| | | (c) Consider use of a flow diagram **Figure** |
| Descriptive data | 14* | (a) Give characteristics of study participants (eg demographic, clinical, social) and information on exposures and potential confounders **Page 11 and Table 1** |
| | | (b) Indicate number of participants with missing data for each variable of interest **Figure** |
| | | (c) Summarise follow-up time (eg, average and total amount) **Page 11** |
| Outcome data | 15* | Report numbers of outcome events or summary measures over time **Tables 2 and 4** |
| Main results | 16 | (*a*) Give unadjusted estimates and, if applicable, confounder-adjusted estimates and their precision (eg, 95% confidence interval). Make clear which confounders were adjusted for and why they were included **Pages 11 to 13 and Tables 2 to 4** |
| | | (*b*) Report category boundaries when continuous variables were categorized **Page 9** |
| | | (*c*) If relevant, consider translating estimates of relative risk into absolute risk for a meaningful time period **Pages 11 to 13 and Table 2** |
| Other analyses | 17 | Report other analyses done—eg analyses of subgroups and interactions, and sensitivity analyses **Pages 11 to 13 and Table 4** |
| **Discussion** | | |
| Key results | 18 | Summarise key results with reference to study objectives **Page 14** |
| Limitations | 19 | Discuss limitations of the study, taking into account sources of potential bias or imprecision. Discuss both direction and magnitude of any potential bias **Page 14** |
| Interpretation | 20 | Give a cautious overall interpretation of results considering objectives, limitations, multiplicity of analyses, results from similar studies, and other relevant evidence **Pages 14 to 15** |
| Generalisability | 21 | Discuss the generalisability (external validity) of the study results **Page 15** |
| **Other information** | | |
| Funding | 22 | Give the source of funding and the role of the funders for the present study and, if applicable, for the original study on which the present article is based **Page 16** |

*Give information separately for exposed and unexposed groups.

For peer review only

**Note:** An Explanation and Elaboration article discusses each checklist item and gives methodological background and published examples of transparent reporting. The STROBE checklist is best used in conjunction with this article (freely available on the Web sites of PLoS Medicine at http://www.plosmedicine.org/, Annals of Internal Medicine at http://www.annals.org/, and Epidemiology at http://www.epidem.com/). Information on the STROBE Initiative is available at http://www.strobe-statement.org.

For peer review only


For peer review only - http://bmjopen.bmj.com/site/about/guidelines.xhtml

# Mortality in gastro-oesophageal reflux disease in a population-based nationwide cohort study of Swedish twins

| | |
|---|---|
| Journal: | *BMJ Open* |
| Manuscript ID | bmjopen-2020-037456.R1 |
| Article Type: | Original research |
| Date Submitted by the Author: | 23-Apr-2020 |
| Complete List of Authors: | Ness-Jensen, Eivind; Karolinska Institute, Upper Gastrointestinal Research, Department of Molecular Medicine and Surgery; NTNU, HUNT Research Centre, Department of Public Health and Nursing<br>Santoni, Giola; Karolinska Institute, Upper Gastrointestinal Research, Department of Molecular Medicine and Surgery<br>Gottlieb-Vedi, Eivind; Karolinska Institute, Upper Gastrointestinal Research, Department of Molecular Medicine and Surgery<br>Lindam, Anna; Karolinska Institute, Upper Gastrointestinal Research, Department of Molecular Medicine and Surgery<br>Pedersen, Nancy; Karolinska Institute, Department of Medical Epidemiology and Biostatistics<br>Lagergren, Jesper; Karolinska Institute, Department of Molecular medicine and Surgery; King's College London, Division of Cancer Studies |
| <b>Primary Subject Heading</b>: | Gastroenterology and hepatology |
| Secondary Subject Heading: | Epidemiology |
| Keywords: | EPIDEMIOLOGY, Oesophageal disease < GASTROENTEROLOGY, Gastrointestinal tumours < ONCOLOGY |
| | |

SCHOLARONE™
Manuscripts

*I, the Submitting Author has the right to grant and does grant on behalf of all authors of the Work (as defined in the below author licence), an exclusive licence and/or a non-exclusive licence for contributions from authors who are: i) UK Crown employees; ii) where BMJ has agreed a CC-BY licence shall apply, and/or iii) in accordance with the terms applicable for US Federal Government officers or employees acting as part of their official duties; on a worldwide, perpetual, irrevocable, royalty-free basis to BMJ Publishing Group Ltd ("BMJ") its licensees and where the relevant Journal is co-owned by BMJ to the co-owners of the Journal, to publish the Work in this journal and any other BMJ products and to exploit all rights, as set out in our licence.*

*The Submitting Author accepts and understands that any supply made under these terms is made by BMJ to the Submitting Author unless you are acting as an employee on behalf of your employer or a postgraduate student of an affiliated institution which is paying any applicable article publishing charge ("APC") for Open Access articles. Where the Submitting Author wishes to make the Work available on an Open Access basis (and intends to pay the relevant APC), the terms of reuse of such Open Access shall be governed by a Creative Commons licence – details of these licences and which Creative Commons licence will apply to this Work are set out in our licence referred to above.*

*Other than as permitted in any relevant BMJ Author's Self Archiving Policies, I confirm this Work has not been accepted for publication elsewhere, is not being considered for publication elsewhere and does not duplicate material already published. I confirm all authors consent to publication of this Work and authorise the granting of this licence.*

For peer review only

**Title: Mortality in gastro-oesophageal reflux disease in a population-based nationwide cohort study of Swedish twins**

**Authors:** Eivind Ness-Jensen, MD, PhD[1,2,3], Giola Santoni, PhD[1], Eivind Gottlieb-Vedi, MD[1], Anna Lindam, PhD[1], Nancy L. Pedersen, PhD[4], Jesper Lagergren, MD, PhD[1,5]

**Affiliations and addresses:** [1]Upper Gastrointestinal Surgery, Department of Molecular medicine and Surgery, Karolinska Institutet, Karolinska University Hospital, S-171 76 Stockholm, Sweden;

[2]HUNT Research Centre, Department of Public Health and Nursing, NTNU, Norwegian University of Science and Technology, Forskningsvegen 2, N-7600 Levanger, Norway;

[3]Medical Department, Levanger Hospital, Nord-Trøndelag Hospital Trust, P.O. Box 333, N-7601 Levanger, Norway;

[4]Department of Medical Epidemiology and Biostatistics, Karolinska Institutet, S-171 76 Stockholm, Sweden;

[5]School of Cancer and Pharmaceutical Sciences, King's College London, London SE1 9RT, United Kingdom

**Corresponding author:** Eivind Ness-Jensen, eivind.ness-jensen@ki.se, Upper Gastrointestinal Surgery, Department of Molecular medicine and Surgery, Karolinska Institutet, Karolinska University Hospital, S-171 76 Stockholm, Sweden. Telephone: 0046-851776012

**Word count:** 2604

For peer review only



For peer review only - http://bmjopen.bmj.com/site/about/guidelines.xhtml

## Abstract

**Objectives:** The public health disorder gastro-oesophageal reflux disease (GORD) is linked with several comorbidities, including oesophageal adenocarcinoma, but whether life expectancy is reduced by GORD is uncertain. This study assessed all-cause and cancer-specific mortality in GORD after controlling for confounding by heredity and other factors.

**Design:** Population-based cohort study from 1998 through 2015.

**Setting:** Swedish nationwide study.

**Participants:** Twins (n=40,961) born in 1958 or earlier in Sweden.

**Exposure:** GORD symptoms reported in structured computer-assisted telephone interviews.

**Outcomes:** The primary outcome was all-cause mortality and the secondary outcome was cancer-specific mortality among twins with GORD and twins without GORD. Hazard ratios (HR) and 95% confidence intervals (CI) were analysed using parametric survival models, both in individual twin analyses and co-twin pair analyses, with adjustment for body mass index, smoking, education and comorbidity.

**Results:** Among 40,961 individual twins, 5,812 (14.2%) had GORD at baseline and 8,062 (19.7%) died during follow-up of up to 16 years. The risk of all-cause mortality (HR=1.00, 95% CI 0.94-1.07) and cancer-specific mortality (HR=0.99, 95% CI 0.89-1.10) were not increased in individual twins with GORD compared to individual twins without GORD. Similarly, there were no differences in mortality outcomes in within-pair analyses. The oesophageal adenocarcinoma-specific mortality rate was 0.45 (95% CI 0.32-0.66) per 1,000 person-years in individual twins with GORD and 0.22 (95% CI 0.18-0.27) per 1,000 person-years without GORD, rendering an adjusted HR of 2.01 (95% CI 1.35-2.98).


For peer review only

**Conclusions:** GORD did not increase all-cause or cancer-specific mortality when taking heredity and other confounders into account. The increased relative risk of mortality in oesophageal adenocarcinoma was low in absolute numbers.

**Keywords:** Survival; prognosis, heartburn; neoplasm; heredity; comorbidity

For peer review only



For peer review only - http://bmjopen.bmj.com/site/about/guidelines.xhtml

## Article Summary

### Strengths and limitations of this study

- The twin design which adjusts for heredity and shared familial confounders
- The prospective and nationwide population-based approach which counteracts recall and selection bias, as well as chance errors
- Valid and complete long-term follow-up using national registers
- Assessment of potential confounders
- No objective assessment of gastro-oesophageal reflux disease

For peer review only



For peer review only - http://bmjopen.bmj.com/site/about/guidelines.xhtml

## Introduction

Gastro-oesophageal reflux disease (GORD) is defined by troublesome heartburn and acid regurgitation occurring at least weekly or GORD-specific complications.[1] GORD affects between 10-30% of adults in the Western world and is one of the most common reasons for visits to gastroenterologists and general practitioners.[2, 3] Heredity, obesity, and tobacco smoking are the only established risk factors, while socioeconomic factors (mainly educational level) might also influence the risk of GORD.[4-7] Twin studies have shown that the heritability for GORD is 31-43%.[8, 9] Because GORD is associated with several conditions, e.g. cardiovascular disease, various gastrointestinal symptoms, anxiety, depression, sleep disorders,[10-13] reductions in health-related quality of life,[14, 15] and oesophageal and gastric cardia adenocarcinoma,[16] it has been hypothesised that GORD reduces life expectancy in general and increases mortality from cancer specifically. This is an important topic, not the least considering the high prevalence of GORD, and the consequences any influence on life expectancy would mean for healthcare and public health interventions. However, the research that has examined whether GORD increases the risk of mortality has been limited and provided conflicting results, some indicating a reduced survival and other not.[17-20] No previous study has taken influence of all risk factors for GORD into account as confounders, particularly not heredity or shared familial exposures.

The present study aimed to clarify whether GORD influences the mortality for all causes, cancer in general, and oesophageal adenocarcinoma in specific by conducting a large and comprehensive twin study, controlling for genetic and familial influences, together with other potential confounders.



For peer review only - http://bmjopen.bmj.com/site/about/guidelines.xhtml

## Methods

Study design

This population-based twin study was based on data from the Swedish Twin Registry, during the study period 1998 through 2015. This Swedish Twin registry incorporates comprehensive data retrieved directly from twins combined with data collected from Swedish national health registries. The personal identity number, which is assigned to each Swedish inhabitant, enabled exact linkage of participants' data between the data sources.[21] The study was approved by the Regional Ethical Review Board in Stockholm reference number 2010/582-31/1). All twins gave a broad informed consent for data collection and research when participating. The study protocol conforms to the ethical guidelines of the 1975 Declaration of Helsinki as reflected in a priori approval by the institution's human research committee. Patients or the public were not involved in the design, or conduct, or reporting, or dissemination plans of our research.

Cohort

The study cohort was based on data from the Swedish Twin Registry, the largest and most comprehensive twin registry globally.[22, 23] It was established in the late 1950s and includes virtually all twins born in Sweden from 1886 onwards. During 1998-2002, the Screening Across the Lifespan Twin study (SALT) was performed with structured computer-assisted telephone interviews of twins born in 1958 or earlier and recorded in the Twin Registry, including assessment of GORD symptoms and risk factors for GORD.[22, 23] Data from the SALT interviews were used to define the study cohort and to assess information about GORD and



For peer review only - http://bmjopen.bmj.com/site/about/guidelines.xhtml

For peer review only

the potential confounders heredity, body mass index (BMI), tobacco smoking, and education. Zygosity was assessed by a separate questionnaire sent to the twins. The twins were defined as monozygotic if both twins in a same-sexed pair reported they were "alike as two peas in a pod" and as dizygotic if they reported to be "not more alike than siblings". This simple method has been shown to be 99% accurate in of determining zygosity compared to DNA-testing.[22] The Swedish Twin Registry is regularly updated with information from other nationwide Swedish registries, i.e. the Cause of Death Registry, Cancer Registry and Patient Registry, which are briefly presented below.

*The Swedish Cause of Death Registry* provided data on all-cause and cancer-specific mortality. This registry includes date of death and causes of death for all Swedish residents since 1961, regardless of whether they died in Sweden or abroad. The information about date of death and cause of death is 100% and 99% complete, respectively.[24, 25]

*The Swedish Cancer Registry* had information about the histological type of oesophageal cancer (adenocarcinoma). This registry started in 1958 and includes standardized records of all newly diagnosed malignancies in Sweden, including date of diagnosis, tumour site, and histological type. Histological type is registered in accordance with the World Health Organization's classification of histology (C24). The general completeness of the registry is 96% and it is 98% complete regarding recording of oesophageal adenocarcinoma, and for these patients, the histological verification is 100% complete.[26, 27]

*The Swedish Patient Registry* contained data on comorbidity. The registry contains date and International Classification of Diseases (ICD) versions 9 and 10 codes of diagnoses from all



For peer review only - http://bmjopen.bmj.com/site/about/guidelines.xhtml

inpatient healthcare in Sweden from 1987 onwards and all specialist outpatient healthcare since 2001. This registry has a positive predictive value of any primary diagnosis close to 100%.[28] Diagnoses registered three years before and three years after the SALT interviews were included in the assessment of comorbidity. This restriction in time was done to counteract misclassification of comorbidity due to different lengths of follow-up among the participating twins.

Exposure

The twins were defined as being exposed to GORD if they reported in the SALT interview to have: 1) heartburn at least weekly, 2) regurgitation at least weekly, or 3) retrosternal pain at least weekly combined with antacid relief.[1]

Outcomes

The main outcome was all-cause mortality, which included any deaths, regardless of cause. A secondary outcome was overall cancer-specific mortality, which included deaths related to any cancer (ICD-7 140-199 or ICD-10 C00-C97), excluding non-melanoma skin cancer (ICD-7 191 or ICD-10 C44). The other secondary outcome was oesophageal adenocarcinoma-specific mortality, defined as deaths related to oesophageal or gastroesophageal junctional adenocarcinoma (ICD-7 150 or 151.1 and C24 096 or ICD-10 C15 or C16.0 and C24 096).

Confounders

Data on BMI, tobacco smoking, and education were retrieved from the SALT interviews. BMI was calculated as the weight (kilograms) divided by the square height (meters). Smoking status included consumption of cigarettes, cigars, and pipe. The level of education was



For peer review only - http://bmjopen.bmj.com/site/about/guidelines.xhtml

assessed by the highest reported completed education qualification. Data on comorbidity were collected from the Swedish Patient Registry. The Royal College of Surgeons version of the Charlson comorbidity index was used to define and classify comborbidity.[29] This is the recommended version for registry-based research.[30]

Statistical analyses

Mortality rates per 1,000 person-years were compared between individuals with and without GORD for all three mortality outcomes. Parametric survival models with Weibull distribution and sandwich estimator for the variance clustered by the twins' pair identity were used to calculate hazard ratios (HR) with 95% confidence intervals (CI). These models correct for within twin pair dependency and to help avoid underestimation of the variance. The baseline hazard was modelled with a linear and a quadratic time term. Proportionality of the hazards was verified in all analyses. Time at risk was defined from the date of the SALT interview (1998-2002), i.e., when GORD was assessed, until the date of death or the end of the study period (December 31, 2015).

The mortality among twins with GORD was compared with the mortality among twins without GORD in a stepwise series of analyses. First, external control analyses were performed using all individual twins, comparing individual twins with GORD to individual twins without GORD. Second, within-pair co-twin analyses of dizygotic twins discordant for GORD were performed. Third, within-pair co-twin analyses of monozygotic twins discordant for GORD were conducted. In the two latter analyses, only complete twin pairs were included. These three analysis steps were performed for each mortality outcome.



For peer review only - http://bmjopen.bmj.com/site/about/guidelines.xhtml

Stepwise adjustments for confounders were performed. First, a basic model adjusted for age (continuous) and sex. Second, the results were additionally adjusted for BMI (categorised into <25, 25-30, or >30), smoking (never, former, or current), and years of completed education (0-9.5 years, 9.5-12.5 years, or >12.5 years). Third, the results were further adjusted for comorbidity (Charlson comorbidity index score 0, 1, or ≥2),[29] which was done to assess whether comorbidity could explain any association between GORD and mortality.

In order to examine effect modification, analyses were stratified by age (≤60 or >60 years) and sex (except for the monozygotic twin analyses). In the monozygotic twin analysis of men aged 40-60 years, the HRs were estimated with exponential distribution and sandwich estimator for the variance, clustered by the twins' pair identity in order for the model to converge. This result should be similar to the model with Weibull distribution, which did not converge in this analysis.

A senior biostatistician (GS) conducted the data management and statistical analysis following a pre-defined study protocol. The statistical analyses were performed using Stata MP version 15, StataCorp LP, College Station, TX, USA.

For peer review only

## Results

Participants

Among 43,350 individual twins who participated in SALT, 40,961 (95.5%) answered the questions relevant for the present study and were thus included in the final analysis. A flowchart describing the study cohort is shown in Figure 1. Among the participating twins, 8,062 (19.7%) died during follow-up of up to 16 years, including 2,845 (6.9%) from any cancer and 127 (0.3%) from oesophageal adenocarcinoma. Characteristics of the included twins with and without GORD are shown in Table 1. The median age was 56 years in both groups. In all, 14.2% had GORD and GORD was similarly common in both sexes and in both dizygotic and monozygotic twins. Compared to twins without GORD, the twins with GORD were more often overweight or obese, tobacco smokers, less educated, and diagnosed with comorbidities (Table 1). The study included 2,501 dizygotic twin pairs discordant for GORD and 749 monozygotic twin pairs discordant for GORD.

Mortality from any cause

The all-cause mortality rate of all individual twins was 16.2 (95% CI 15.3-17.2) per 1,000 person-years in twins with GORD and also 16.2 (95% CI 15.8-16.7) per 1,000 person-years in twins without GORD (Table 2). In dizygotic twin pairs discordant for GORD, the all-cause mortality rates were 13.3 (95% CI 12.1-14.7) per 1,000 person-years in twins with GORD and 13.3 (95% CI 12.2-14.7) per 1,000 person-years for their co-twins without GORD. In monozygotic twin pairs discordant for GORD, the all-cause mortality rates were 12.0 (95% CI 10.0-14.4) per 1,000 person-years in twins with GORD and 12.2 (95% CI 10.2-14.6) per 1,000 person-years in their co-twins without GORD.

For peer review only

The fully adjusted HR of all-cause mortality was 1.00 (95% CI 0.94-1.07) comparing all individual twins with GORD to individual twins without GORD (Table 3). In the dizygotic twin analysis, the corresponding HR was 0.99 (95% CI 0.87-1.14). In the monozygotic twin analysis, the adjusted HR was 1.11 (95% CI 0.87-1.40). The analyses stratified by sex and age showed similar HRs without any association between GORD and all-cause mortality (Table 4).

<u>Mortality from any cancer</u>

The overall cancer-specific mortality rate of all individual twins was 5.8 (95% CI 5.3-6.4) per 1,000 person-years in those with GORD and 5.7 (95% CI 5.5-6.0) per 1,000 person-years in those without GORD (Table 2). The dizygotic twin analysis also showed similar cancer-specific mortality rates in twins with GORD (4.9 [95% CI 4.2-5.7] per 1,000 person-years) and their co-twin without (4.9 8 [95% CI 4.2-5.7] per 1,000 person-years). In the monozygotic twin analysis, the corresponding rates were 5.2 (95% CI 3.9-6.9) per 1,000 person-years in twins with GORD and 4.6 (95% CI 3.5-6.3) per 1,000 person-years in their co-twins with no GORD.

The fully adjusted HR of overall cancer-specific mortality was 0.99 (95% CI 0.89-1.10) comparing all individual twins with GORD to individual twins without GORD (Table 3). The corresponding HRs in dizygotic twins and monozygotic twins were 0.99 (95% CI 0.78-1.24) and 1.28 (95% CI 0.87-1.87), respectively. The analyses stratified by sex and age showed similar HRs and no association between GORD and overall cancer-specific mortality (Table 4).



For peer review only - http://bmjopen.bmj.com/site/about/guidelines.xhtml

Mortality from oesophageal adenocarcinoma

The oesophageal adenocarcinoma-specific mortality rate was 0.45 (95% CI 0.32-0.66) per 1,000 person-years in all individual twins with GORD, compared to 0.22 (95% CI 0.18-0.27) per 1,000 person-years in twins without GORD (Table 2). In dizygotic twins, this rate was 0.39 (95% CI 0.23-0.74) per 1,000 person-years in the twins with GORD and 0.26 (95% CI 0.13-0.58) per 1,000 person-years in the twins without GORD. The mortality rate was 0.32 (95% CI 0.10-1.58) per 1,000 person-years in the monozygotic twins with GORD, while there was no oesophageal adenocarcinoma-specific mortality in the monozygotic twins without GORD.

The fully adjusted HR was 2.01 (95% CI 1.35-2.98) for oesophageal adenocarcinoma-specific mortality comparing all individual twins with GORD to those without GORD (Table 3). In dizygotic twins, the corresponding HR was 1.44 (95% CI 0.60-3.45), while the statistical power was insufficient for monozygotic twin analysis. The HR was 3.71 (95% CI 1.90-7.28) in men aged 40-60 years, and 1.60 (95% CI 0.77-3.32) in men aged >60 years (Table 4). The stratified dizygotic twin analyses had low statistical power, but the fully adjusted HR for oesophageal adenocarcinoma-specific mortality was 2.07 (95% CI 0.53-8.08) among men aged 40-60 years and 0.82 (95% CI 0.15-4.61) among men aged >60 years.



For peer review only - http://bmjopen.bmj.com/site/about/guidelines.xhtml

## Discussion

This large-scale twin study found no increased all-cause or cancer-specific mortality in twins with GORD compared to twins without GORD. The risk of mortality in oesophageal adenocarcinoma was higher in twins with GORD than in twins without GORD, but the absolute risk was still low.

Among methodological strengths is the twin design, which enabled the first study on the topic with adjustment for heredity and shared familial confounders. The prospective and nationwide population-based approach counteracted recall and selection bias, as well as chance errors. The high-quality and complete data reduced misclassification and enabled long and complete follow-up of all participants. The assessment of mortality was valid and complete. The definition of GORD was the evidence-based Montreal consensus, which remains the definition of choice for research purposes.[1] The prevalence of GORD in this study coincides well with the prevalence reported in similar Western populations,[2] indicating validity of the definition of GORD. The assessment of potential confounders through the structured SALT interviews (BMI, tobacco smoking, and education) and the Patient Registry (comorbidity) allowed for adjustment of all risk factors for GORD and mortality, i.e. all plausible confounders. Yet, a weakness is residual confounding, which cannot be entirely ruled out in this observational study. The rate of missing values for the variables included in the study was low, and all analyses were complete case analyses. The large sample size allowed for age and sex stratified analyses to assess effect modification with age and sex, but the dizygotic and monozygotic co-twin analyses had limited statistical power, although the results generally supported the overall findings.



For peer review only - http://bmjopen.bmj.com/site/about/guidelines.xhtml

The results of the present study showing no increased all-cause mortality in individuals with GORD corroborates the findings of our recent cohort study from Norway,[20] a cohort study from the United States,[18] and a cohort study from Iran.[19] However, three cohort studies from the United Kingdom showed a 1.16- to 1.6-fold increase in mortality in people with GORD compared with the background population, the majority of deaths being due to cardiac disease.[17] The increased mortality found in some studies could be due to prevalent cancers provoking GORD symptoms. No earlier study has heredity as a confounder, although heredity is a strong risk factor for GORD.[8]

GORD is common in Western populations, with 10-30% prevalence in adults [2, 3]. The present study implies that individuals with GORD do not need to worry about any increased risk of dying. The increased risk of death from oesophageal adenocarcinoma should not be overemphasized because the absolute risk is still low even in the presence of GORD. However, if the incidence of oesophageal adenocarcinoma continues to increase strongly without any improvements in the survival, the influence of mortality from this tumour could increase.

In conclusion, this nationwide Swedish population-based cohort study in twins with long and complete follow-up and adjustment for confounders indicates that GORD does not increase the risk of all-cause or cancer-specific mortality. Despite the increased relative risk of mortality from oesophageal adenocarcinoma in individuals with GORD, the absolute risk is still low.



For peer review only - http://bmjopen.bmj.com/site/about/guidelines.xhtml

**Acknowledgments**

We are grateful to the Swedish Twin Registry for access to data. This Registry is managed by Karolinska Institutet and receives funding from the Swedish Research Council (grant 2017-00641).

**Disclosure of interest**

The authors report no conflict of interest.

**Funding**

This work was supported by the Swedish Research Council (SIMSAM) under grant number 2017-00641; Swedish Society of Medicine; and the United European Gastroenterology (UEG Research Prize to JL). The funders did not have any role in the study design, in the collection, analysis, or interpretation of data.

**Data availability statement**

The data set used in this paper is available through application to The Swedish Twin Registry (https://ki.se/en/research/swedish-twin-registry-for-researchers)

**Author statement**

(i) Guarantor of the article: Eivind Ness-Jensen; (ii) Specific author contributions: **ENJ:** study concept and design; acquisition of data; analysis and interpretation of data; drafting of the manuscript; critical revision of the manuscript for important intellectual content; obtained funding. **GS:** study concept and design; analysis and interpretation of data; critical revision of the manuscript for important intellectual content; statistical analysis. **EGV:** study concept



For peer review only - http://bmjopen.bmj.com/site/about/guidelines.xhtml

For peer review only

and design; acquisition of data; analysis and interpretation of data; critical revision of the manuscript for important intellectual content. **AL:** study concept and design; acquisition of data; analysis and interpretation of data; critical revision of the manuscript for important intellectual content. **NP:** study concept and design; acquisition of data; analysis and interpretation of data; critical revision of the manuscript for important intellectual content. **JL:** study concept and design; analysis and interpretation of data; critical revision of the manuscript for important intellectual content; obtained funding; (iii) All authors approved the final version of the manuscript.

For peer review only

## References


1. Vakil N, van Zanten SV, Kahrilas P, et al. The Montreal definition and classification of gastroesophageal reflux disease: a global evidence-based consensus. Am J Gastroenterol 2006;101:1900-20; quiz 1943.
2. El-Serag HB, Sweet S, Winchester CC, et al. Update on the epidemiology of gastro-oesophageal reflux disease: a systematic review. Gut 2014;63:871-80.
3. Ness-Jensen E, Lindam A, Lagergren J, et al. Changes in prevalence, incidence and spontaneous loss of gastro-oesophageal reflux symptoms: a prospective population-based cohort study, the HUNT study. Gut 2012;61:1390-7.
4. Dent J, El-Serag HB, Wallander MA, et al. Epidemiology of gastro-oesophageal reflux disease: a systematic review. Gut 2005;54:710-7.
5. Nilsson M, Johnsen R, Ye W, et al. Lifestyle related risk factors in the aetiology of gastro-oesophageal reflux. Gut 2004;53:1730-5.
6. Jansson C, Nordenstedt H, Johansson S, et al. Relation between gastroesophageal reflux symptoms and socioeconomic factors: a population-based study (the HUNT Study). Clin Gastroenterol Hepatol 2007;5:1029-34.
7. Hallan A, Bomme M, Hveem K, et al. Risk factors on the development of new-onset gastroesophageal reflux symptoms. A population-based prospective cohort study: the HUNT study. Am J Gastroenterol 2015;110:393-400; quiz 401.
8. Cameron AJ, Lagergren J, Henriksson C, et al. Gastroesophageal reflux disease in monozygotic and dizygotic twins. Gastroenterology 2002;122:55-9.
9. Mohammed I, Cherkas LF, Riley SA, et al. Genetic influences in gastro-oesophageal reflux disease: a twin study. Gut 2003;52:1085-9.
10. Jansson C, Nordenstedt H, Wallander MA, et al. Severe gastro-oesophageal reflux symptoms in relation to anxiety, depression and coping in a population-based study. Aliment Pharmacol Ther 2007;26:683-91.
11. Jansson C, Nordenstedt H, Wallander MA, et al. Severe symptoms of gastro-oesophageal reflux disease are associated with cardiovascular disease and other gastrointestinal symptoms, but not diabetes: a population-based study. Aliment Pharmacol Ther 2008;27:58-65.
12. Lindam A, Jansson C, Nordenstedt H, et al. A population-based study of gastroesophageal reflux disease and sleep problems in elderly twins. PLoS One 2012;7:e48602.
13. Lindam A, Ness-Jensen E, Jansson C, et al. Gastroesophageal Reflux and Sleep Disturbances: A Bidirectional Association in a Population-Based Cohort Study, The HUNT Study. Sleep 2016;39:1421-7.
14. Wiklund I, Carlsson J, Vakil N. Gastroesophageal reflux symptoms and well-being in a random sample of the general population of a Swedish community. Am J Gastroenterol 2006;101:18-28.
15. Ronkainen J, Aro P, Storskrubb T, et al. Gastro-oesophageal reflux symptoms and health-related quality of life in the adult general population--the Kalixanda study. Aliment Pharmacol Ther 2006;23:1725-33.
16. Lagergren J, Bergstrom R, Lindgren A, et al. Symptomatic gastroesophageal reflux as a risk factor for esophageal adenocarcinoma. N Engl J Med 1999;340:825-31.
17. Becher A, El-Serag HB. Mortality associated with gastroesophageal reflux disease and its non-malignant complications: a systematic review. Scand J Gastroenterol 2008;43:645-53.
18. Talley NJ, Locke GR, 3rd, McNally M, et al. Impact of gastroesophageal reflux on survival in the community. Am J Gastroenterol 2008;103:12-9.
19. Islami F, Pourshams A, Nasseri-Moghaddam S, et al. Gastroesophageal Reflux Disease and overall and Cause-specific Mortality: A Prospective Study of 50000 Individuals. Middle East J Dig Dis 2014;6:65-80.




For peer review only - http://bmjopen.bmj.com/site/about/guidelines.xhtml

20. Ness-Jensen E, Gottlieb-Vedi E, Wahlin K, et al. All-cause and cancer-specific mortality in GORD in a population-based cohort study (the HUNT study). Gut 2018;67:209-215.
21. Ludvigsson JF, Otterblad-Olausson P, Pettersson BU, et al. The Swedish personal identity number: possibilities and pitfalls in healthcare and medical research. Eur J Epidemiol 2009;24:659-67.
22. Lichtenstein P, De Faire U, Floderus B, et al. The Swedish Twin Registry: a unique resource for clinical, epidemiological and genetic studies. J Intern Med 2002;252:184-205.
23. Lichtenstein P, Sullivan PF, Cnattingius S, et al. The Swedish Twin Registry in the third millennium: an update. Twin Res Hum Genet 2006;9:875-82.
24. Johansson LA, Westerling R. Comparing Swedish hospital discharge records with death certificates: implications for mortality statistics. Int J Epidemiol 2000;29:495-502.
25. Brooke HL, Talback M, Hornblad J, et al. The Swedish cause of death register. Eur J Epidemiol 2017;32:765-773.
26. Barlow L, Westergren K, Holmberg L, et al. The completeness of the Swedish Cancer Register: a sample survey for year 1998. Acta Oncol 2009;48:27-33.
27. Lindblad M, Ye W, Lindgren A, et al. Disparities in the classification of esophageal and cardia adenocarcinomas and their influence on reported incidence rates. Ann Surg 2006;243:479-85.
28. Ludvigsson JF, Andersson E, Ekbom A, et al. External review and validation of the Swedish national inpatient register. BMC Public Health 2011;11:450.
29. Armitage JN, van der Meulen JH, Royal College of Surgeons Co-morbidity Consensus G. Identifying co-morbidity in surgical patients using administrative data with the Royal College of Surgeons Charlson Score. Br J Surg 2010;97:772-81.
30. Brusselaers N, Lagergren J. The Charlson Comorbidity Index in Registry-based Research. Methods Inf Med 2017;56:401-406.

For peer review only

**Table 1.** Baseline characteristics of twins with and without gastro-oesophageal reflux disease (GORD)

| | **GORD** | | **No GORD** | |
|---|---|---|---|---|
| | Number (%) | | Number (%) | |
| Total | 5,812 | (14.2) | 35,149 | (85.8) |
| Age, years* | 56 | (41-95) | 56 | (41-99) |
| Sex | | | | |
| Men | 2,673 | (46.0) | 16,683 | (47.5) |
| Women | 3,139 | (54.0) | 18,466 | (52.5) |
| Zygosity | | | | |
| Monozygotic | 1,444 | (24-8) | 8,860 | (25.2) |
| Dizygotic | 4,368 | (75.2) | 26,289 | (75.8) |
| BMI | | | | |
| <25 | 2,568 | (44.2) | 19,577 | (55.7) |
| 25-30 | 2,535 | (43.6) | 12,909 | (36.7) |
| >30 | 709 | (12.2) | 2,663 | (7.6) |
| Tobacco smoking-status | | | | |
| Never | 1,236 | (21.3) | 8,985 | (25.6) |
| Former | 3,330 | (57.3) | 19,104 | (54.4) |
| Current | 1,246 | (21.4) | 7,060 | (20.1) |
| Education, years | | | | |
| 0-9.5 | 3,019 | (51.9) | 16,405 | (46.7) |
| 9.5-12.5 | 1,625 | (28.0) | 9,895 | (28.2) |
| >12.5 | 1,168 | (20.1) | 1,168 | (25.2) |
| Charlson co-morbidity index | | | | |
| 0 | 5,190 | (89.3) | 31,820 | (90.5) |
| 1 | 524 | (9.0) | 2,883 | (8.2) |
| >1 | 98 | (1.7) | 446 | (1.3) |

*Median (range)

For peer review only

**Table 2.** Number of deaths and mortality rates for all-cause, cancer-specific, and oesophageal adenocarcinoma (OAC)-specific mortality in twins with and without gastro-oesophageal reflux disease (GORD)

| | Alive (number) | | Deaths (number) | | Mortality rates per 1,000 person-years (95% CI) | |
|---|---|---|---|---|---|---|
| **Outcome** | **GORD** | **No GORD** | **GORD** | **No GORD** | **GORD** | **No GORD** |
| All-cause mortality | | | | | | |
| All twins | 6,922 | 28,227 | 1,140 | 4,672 | 16.2 (15.3-17.2) | 16.2 (15.8-16.7) |
| Dizygotic[a] | 2,091 | 2,090 | 410 | 411 | 13.3 (12.1-14.7) | 13.3 (12.2-14.7) |
| Monozygotic[a] | 638 | 636 | 111 | 113 | 12.0 (10.0-14.4) | 12.2 (10.2-14.6) |
| Overall cancer-specific mortality | | | | | | |
| All twins | 5,404 | 32,707 | 408 | 2437 | 5.8 (5.3-6.4) | 5.7 (5.5-6.0) |
| Dizygotic[a] | 2,351 | 2,351 | 150 | 150 | 4.9 (4.2-5.7) | 4.9 (4.2-5.7) |
| Monozygotic[a] | 701 | 706 | 48 | 43 | 5.2 (3.9-6.9) | 4.6 (3.5-6.3) |
| OAC-specific mortality | | | | | | |
| All twins | 5,780 | 35,049 | 32 | 95 | 0.45 (0.32-0.66) | 0.22 (0.18-0.27) |
| Dizygotic[a] | 2,489 | 2,493 | 12 | 8 | 0.39 (0.23-0.74) | 0.26 (0.13-0.58) |
| Monozygotic[a] | 746 | 749 | 3 | 0 | 0.32 (0.10-1.58) | - |

[a]Discordant for gastro-oesophageal reflux disease

For peer review only

**Table 3.** Hazard ratio (HR) with 95% confidence interval (CI) for all-cause, cancer-specific, and oesophageal adenocarcinoma (OAC)-specific-specific mortality in twins with and without gastro-oesophageal reflux disease

| | Model 1[a] | | | Model 2[b] | | | Model 3[c] | | |
|---|---|---|---|---|---|---|---|---|---|
| **Outcome** | **HR** | **(95% CI)** | | **HR** | **(95% CI)** | | **HR** | **(95% CI)** | |
| All-cause mortality | | | | | | | | | |
| All twins | 1.03 | (0.97 | 1.10) | 1.03 | (0.96 | 1.09) | 1.00 | (0.94 | 1.07) |
| Dizygotic[d] | 0.99 | (0.87 | 1.13) | 1.04 | (0.91 | 1.18) | 0.99 | (0.87 | 1.14) |
| Monozygotic[d] | 0.99 | (0.79 | 1.24) | 1.05 | (0.84 | 1.32) | 1.11 | (0.87 | 1.40) |
| Overall cancer-specific mortality | | | | | | | | | |
| All twins | 1.04 | (0.93 | 1.15) | 1.02 | (0.92 | 1.14) | 0.99 | (0.89 | 1.10) |
| Dizygotic[d] | 1.00 | (0.80 | 1.25) | 1.04 | (0.83 | 1.30) | 0.99 | (0.78 | 1.24) |
| Monozygotic[d] | 1.13 | (0.78 | 1.62) | 1.21 | (0.84 | 1.75) | 1.28 | (0.87 | 1.87) |
| OAC-specific mortality | | | | | | | | | |
| All twins | 2.09 | (1.40 | 3.13) | 2.11 | (1.41 | 3.15) | 2.01 | (1.35 | 2.98) |
| Dizygotic[d] | 1.50 | (0.61 | 3.68) | 1.62 | (0.70 | 3.78) | 1.44 | (0.60 | 3.45) |
| Monozygotic[d] | - | - | - | - | - | - | - | - | - |

[a]Adjusted for age and sex

[b]Adjusted for age, sex, BMI, tobacco smoking status, and education

[c]Adjusted for age, sex, BMI, tobacco smoking status, education, and Charlson comorbidity index

[d]Discordant for gastro-oesophageal reflux disease

For peer review only

**Table 4.** Hazard ratio[a] (HR) with 95% confidence interval (CI) for all-cause, cancer-specific, and oesophageal adenocarcinoma (OAC)-specific mortality in twins with and without gastro-oesophageal reflux disease (GORD)

| | Age 40-60 years | | | | | Age >60 years | | | | |
|---|---|---|---|---|---|---|---|---|---|---|
| | Number of deaths | | | | | Number of deaths | | | | |
| **Outcome** | **GORD** | **No GORD** | **HR** | **(95% CI)** | | **GORD** | **No GORD** | **HR** | **(95% CI)** | |
| **Men** | | | | | | | | | | |
| All-cause mortality | | | | | | | | | | |
| All twins | 140 | 766 | 0.97 | (0.80 | 1.17) | 437 | 2,885 | 0.96 | (0.87 | 1.06) |
| Dizygotic twins[a] | 58 | 54 | 0.96 | (0.66 | 1.39) | 152 | 164 | 0.91 | (0.73 | 1.13) |
| Monozygotic twins[a] | 16 | 16 | 1.09 | (0.57 | 2.15) | 38 | 32 | 1.40 | (0.92 | 2.13) |
| Overall cancer-specific mortality | | | | | | | | | | |
| All twins | 61 | 327 | 0.97 | (0.73 | 1.29) | 151 | 971 | 0.97 | (0.81 | 1.15) |
| Dizygotic twins[a] | 27 | 24 | 1.06 | (0.60 | 1.87) | 51 | 57 | 0.80 | (0.54 | 1.18) |
| Monozygotic twins[a] | 3 | 5 | 0.65 | (0.12 | 3.38)[e] | 20 | 13 | 1.80 | (0.96 | 3.38) |
| OAC-specific mortality | | | | | | | | | | |
| All twins | 14 | 22 | 3.71 | (1.90 | 7.28) | 9 | 36 | 1.60 | (0.77 | 3.32) |
| Dizygotic twins[a] | 6 | 1 | 2.07 | (0.53 | 8.08) | 2 | 3 | 0.82 | (0.15 | 4.61) |
| Monozygotic twins[a] | 1 | 0 | - | - | - | 2 | 0 | - | - | - |
| **Women** | | | | | | | | | | |
| All-cause mortality | | | | | | | | | | |
| All twins | 133 | 676 | 1.03 | (0.85 | 1.26) | 430 | 2,595 | 1.00 | (0.90 | 1.11) |
| Dizygotic twins[a] | 52 | 56 | 1.03 | (0.70 | 1.51) | 148 | 137 | 1.10 | (0.87 | 1.40) |
| Monozygotic twins[a] | 15 | 20 | 0.75 | (0.34 | 1.67) | 42 | 45 | 1.11 | (0.78 | 1.57) |
| Overall cancer-specific mortality | | | | | | | | | | |
| All twins | 65 | 427 | 0.80 | (0.61 | 1.05) | 131 | 712 | 1.10 | (0.91 | 1.33) |
| Dizygotic twins[a] | 23 | 28 | 0.93 | (0.52 | 1.65) | 49 | 41 | 1.30 | (0.84 | 2.03) |
| Monozygotic twins[a] | 11 | 11 | 1.07 | (0.41 | 2.77) | 14 | 14 | 1.06 | (0.52 | 2.15) |
| OAC-specific mortality | | | | | | | | | | |
| All twins | 1 | 9 | 0.51 | (0.06 | 4.09) | 8 | 28 | 1.81 | (0.83 | 3.94) |
| Dizygotic twins[a] | 0 | 0 | - | - | - | 3 | 2 | 1.39 | (0.30 | 6.43) |
| Monozygotic twins[a] | 0 | 0 | - | - | - | 0 | 0 | - | - | - |

[a]Discordant for gastro-oesophageal reflux disease

For peer review only



For peer review only - http://bmjopen.bmj.com/site/about/guidelines.xhtml

**Figure 1.** Study population, sample, and vital status in twins with and without gastro-oesophageal reflux disease (GORD)

*SALT: Screening Across the Lifespan Twin cohort

§OAC: Oesophageal adenocarcinom

For peer review only



For peer review only - http://bmjopen.bmj.com/site/about/guidelines.xhtml

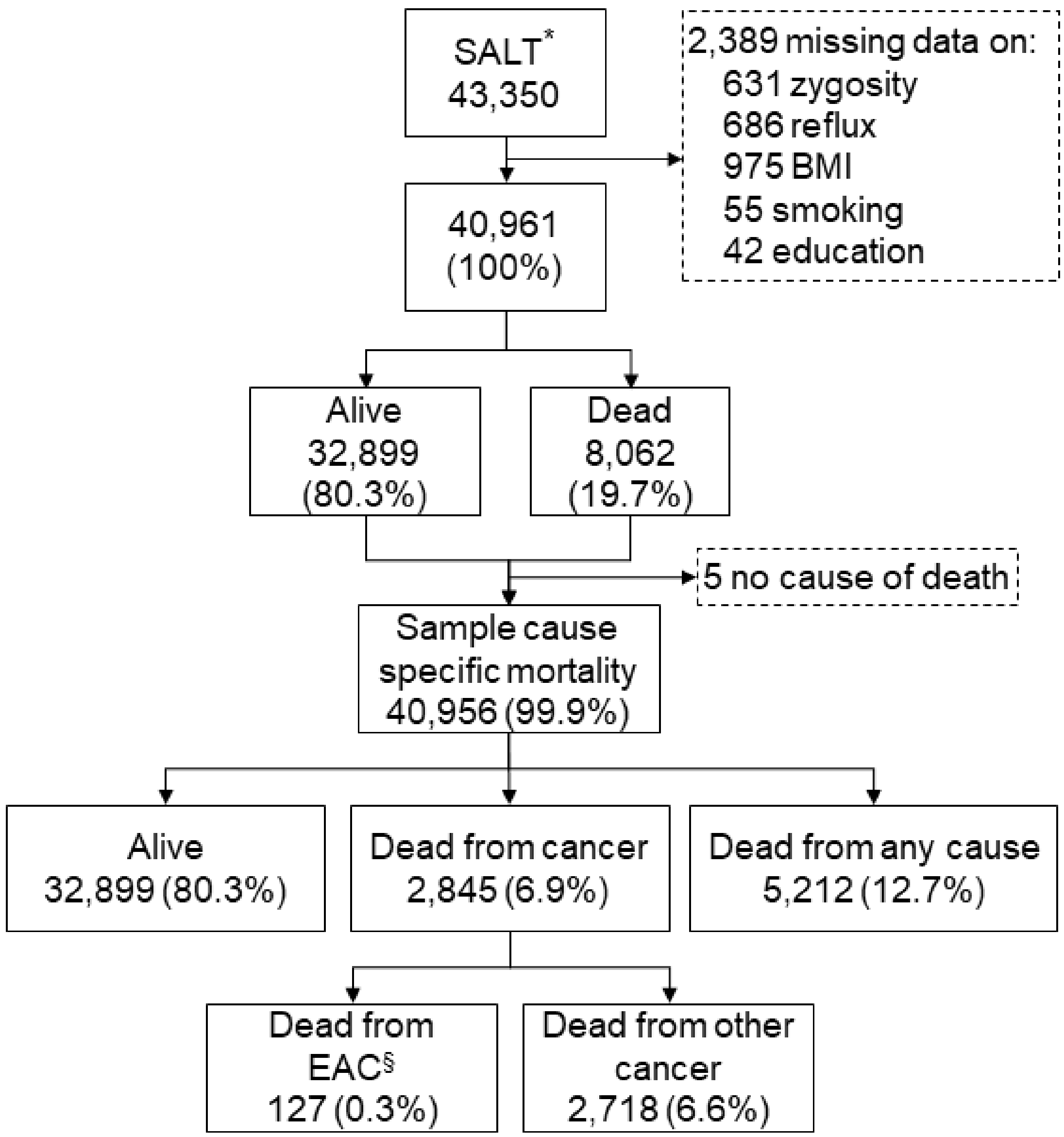
SALT*
43,350
2,389 missing data on:
631 zygosity
686 reflux
975 BMI
55 smoking
42 education
40,961
(100%)
Alive
32,899
(80.3%)
Dead
8,062
(19.7%)
5 no cause of death
Sample cause specific mortality
40,956 (99.9%)
Alive
32,899 (80.3%)
Dead from cancer
2,845 (6.9%)
Dead from any cause
5,212 (12.7%)
Dead from EAC§
127 (0.3%)
Dead from other cancer
2,718 (6.6%)

STROBE Statement—Checklist of items that should be included in reports of ***cohort studies***

| | Item No | Recommendation |
|---|---|---|
| **Title and abstract** | 1 | (*a*) Indicate the study's design with a commonly used term in the title or the abstract **Page 1** |
| | | (*b*) Provide in the abstract an informative and balanced summary of what was done and what was found **Page 2** |
| **Introduction** | | |
| Background/rationale | 2 | Explain the scientific background and rationale for the investigation being reported **Page 5** |
| Objectives | 3 | State specific objectives, including any prespecified hypotheses **Page 5** |
| **Methods** | | |
| Study design | 4 | Present key elements of study design early in the paper **Page 6** |
| Setting | 5 | Describe the setting, locations, and relevant dates, including periods of recruitment, exposure, follow-up, and data collection **Page 6** |
| Participants | 6 | (*a*) Give the eligibility criteria, and the sources and methods of selection of participants. Describe methods of follow-up **Pages 6 to 7** |
| | | (*b*) For matched studies, give matching criteria and number of exposed and unexposed **NA** |
| Variables | 7 | Clearly define all outcomes, exposures, predictors, potential confounders, and effect modifiers. Give diagnostic criteria, if applicable **Pages 6 to 8** |
| Data sources/ measurement | 8* | For each variable of interest, give sources of data and details of methods of assessment (measurement). Describe comparability of assessment methods if there is more than one group **Pages 6 to 8** |
| Bias | 9 | Describe any efforts to address potential sources of bias **Pages 9 to 10** |
| Study size | 10 | Explain how the study size was arrived at **Page 11 and Figure** |
| Quantitative variables | 11 | Explain how quantitative variables were handled in the analyses. If applicable, describe which groupings were chosen and why **Page 9** |
| Statistical methods | 12 | (*a*) Describe all statistical methods, including those used to control for confounding **Pages 9 to 10** |
| | | (*b*) Describe any methods used to examine subgroups and interactions **Pages 9 to 10** |
| | | (*c*) Explain how missing data were addressed **Page 14 and Figure** |
| | | (*d*) If applicable, explain how loss to follow-up was addressed **NA** |
| | | (*e*) Describe any sensitivity analyses |

For peer review only

| | | |
|---|---|---|
| | | **NA** |
| **Results** | | |
| Participants | 13* | (a) Report numbers of individuals at each stage of study—eg numbers potentially eligible, examined for eligibility, confirmed eligible, included in the study, completing follow-up, and analysed<br>**Page 11 and Figure** |
| | | (b) Give reasons for non-participation at each stage<br>**Page 11 and Figure** |
| | | (c) Consider use of a flow diagram<br>**Figure** |
| Descriptive data | 14* | (a) Give characteristics of study participants (eg demographic, clinical, social) and information on exposures and potential confounders<br>**Page 11 and Table 1** |
| | | (b) Indicate number of participants with missing data for each variable of interest<br>**Figure** |
| | | (c) Summarise follow-up time (eg, average and total amount)<br>**Page 11** |
| Outcome data | 15* | Report numbers of outcome events or summary measures over time<br>**Tables 2 and 4** |
| Main results | 16 | (*a*) Give unadjusted estimates and, if applicable, confounder-adjusted estimates and their precision (eg, 95% confidence interval). Make clear which confounders were adjusted for and why they were included<br>**Pages 11 to 13 and Tables 2 to 4** |
| | | (*b*) Report category boundaries when continuous variables were categorized<br>**Page 9** |
| | | (*c*) If relevant, consider translating estimates of relative risk into absolute risk for a meaningful time period<br>**Pages 11 to 13 and Table 2** |
| Other analyses | 17 | Report other analyses done—eg analyses of subgroups and interactions, and sensitivity analyses<br>**Pages 11 to 13 and Table 4** |
| **Discussion** | | |
| Key results | 18 | Summarise key results with reference to study objectives<br>**Page 14** |
| Limitations | 19 | Discuss limitations of the study, taking into account sources of potential bias or imprecision. Discuss both direction and magnitude of any potential bias<br>**Page 14** |
| Interpretation | 20 | Give a cautious overall interpretation of results considering objectives, limitations, multiplicity of analyses, results from similar studies, and other relevant evidence<br>**Pages 14 to 15** |
| Generalisability | 21 | Discuss the generalisability (external validity) of the study results<br>**Page 15** |
| **Other information** | | |
| Funding | 22 | Give the source of funding and the role of the funders for the present study and, if applicable, for the original study on which the present article is based<br>**Page 16** |

*Give information separately for exposed and unexposed groups.

For peer review only

**Note:** An Explanation and Elaboration article discusses each checklist item and gives methodological background and published examples of transparent reporting. The STROBE checklist is best used in conjunction with this article (freely available on the Web sites of PLoS Medicine at http://www.plosmedicine.org/, Annals of Internal Medicine at http://www.annals.org/, and Epidemiology at http://www.epidem.com/). Information on the STROBE Initiative is available at http://www.strobe-statement.org.

For peer review only



For peer review only - http://bmjopen.bmj.com/site/about/guidelines.xhtml

BMJ Open

# Mortality in gastro-oesophageal reflux disease in a population-based nationwide cohort study of Swedish twins


| | |
|---|---|
| Journal: | *BMJ Open* |
| Manuscript ID | bmjopen-2020-037456.R2 |
| Article Type: | Original research |
| Date Submitted by the Author: | 10-Jun-2020 |


| | |
|---|---|
| Complete List of Authors: | Ness-Jensen, Eivind; Karolinska Institute, Upper Gastrointestinal Research, Department of Molecular Medicine and Surgery; NTNU, HUNT Research Centre, Department of Public Health and Nursing<br>Santoni, Giola; Karolinska Institute, Upper Gastrointestinal Research, Department of Molecular Medicine and Surgery<br>Gottlieb-Vedi, Eivind; Karolinska Institute, Upper Gastrointestinal Research, Department of Molecular Medicine and Surgery<br>Lindam, Anna; Karolinska Institute, Upper Gastrointestinal Research, Department of Molecular Medicine and Surgery<br>Pedersen, Nancy; Karolinska Institute, Department of Medical Epidemiology and Biostatistics<br>Lagergren, Jesper; Karolinska Institute, Department of Molecular medicine and Surgery; King's College London, Division of Cancer Studies |


| | |
|---|---|
| <b>Primary Subject Heading</b>: | Gastroenterology and hepatology |
| Secondary Subject Heading: | Epidemiology |
| Keywords: | EPIDEMIOLOGY, Oesophageal disease < GASTROENTEROLOGY, Gastrointestinal tumours < ONCOLOGY |
| | |


SCHOLARONE™ Manuscripts

*I, the Submitting Author has the right to grant and does grant on behalf of all authors of the Work (as defined in the below author licence), an exclusive licence and/or a non-exclusive licence for contributions from authors who are: i) UK Crown employees; ii) where BMJ has agreed a CC-BY licence shall apply, and/or iii) in accordance with the terms applicable for US Federal Government officers or employees acting as part of their official duties; on a worldwide, perpetual, irrevocable, royalty-free basis to BMJ Publishing Group Ltd ("BMJ") its licensees and where the relevant Journal is co-owned by BMJ to the co-owners of the Journal, to publish the Work in this journal and any other BMJ products and to exploit all rights, as set out in our licence.*

*The Submitting Author accepts and understands that any supply made under these terms is made by BMJ to the Submitting Author unless you are acting as an employee on behalf of your employer or a postgraduate student of an affiliated institution which is paying any applicable article publishing charge ("APC") for Open Access articles. Where the Submitting Author wishes to make the Work available on an Open Access basis (and intends to pay the relevant APC), the terms of reuse of such Open Access shall be governed by a Creative Commons licence – details of these licences and which Creative Commons licence will apply to this Work are set out in our licence referred to above.*

*Other than as permitted in any relevant BMJ Author's Self Archiving Policies, I confirm this Work has not been accepted for publication elsewhere, is not being considered for publication elsewhere and does not duplicate material already published. I confirm all authors consent to publication of this Work and authorise the granting of this licence.*

For peer review only

**Title: Mortality in gastro-oesophageal reflux disease in a population-based nationwide cohort study of Swedish twins**

**Authors:** Eivind Ness-Jensen, MD, PhD[1,2,3], Giola Santoni, PhD[1], Eivind Gottlieb-Vedi, MD[1], Anna Lindam, PhD[1], Nancy L. Pedersen, PhD[4], Jesper Lagergren, MD, PhD[1,5]

**Affiliations and addresses:** [1]Upper Gastrointestinal Surgery, Department of Molecular medicine and Surgery, Karolinska Institutet, Karolinska University Hospital, S-171 76 Stockholm, Sweden;

[2]HUNT Research Centre, Department of Public Health and Nursing, NTNU, Norwegian University of Science and Technology, Forskningsvegen 2, N-7600 Levanger, Norway;

[3]Medical Department, Levanger Hospital, Nord-Trøndelag Hospital Trust, P.O. Box 333, N-7601 Levanger, Norway;

[4]Department of Medical Epidemiology and Biostatistics, Karolinska Institutet, S-171 76 Stockholm, Sweden;

[5]School of Cancer and Pharmaceutical Sciences, King's College London, London SE1 9RT, United Kingdom

**Corresponding author:** Eivind Ness-Jensen, eivind.ness-jensen@ki.se, Upper Gastrointestinal Surgery, Department of Molecular medicine and Surgery, Karolinska Institutet, Karolinska University Hospital, S-171 76 Stockholm, Sweden. Telephone: 0046-851776012

**Word count:** 2604

For peer review only

## Abstract

**Objectives:** The public health disorder gastro-oesophageal reflux disease (GORD) is linked with several comorbidities, including oesophageal adenocarcinoma, but whether life expectancy is reduced by GORD is uncertain. This study assessed all-cause and cancer-specific mortality in GORD after controlling for confounding by heredity and other factors.

**Design:** Population-based cohort study from 1998 through 2015.

**Setting:** Swedish nationwide study.

**Participants:** Twins (n=40,961) born in 1958 or earlier in Sweden.

**Exposure:** GORD symptoms reported in structured computer-assisted telephone interviews.

**Outcomes:** The primary outcome was all-cause mortality and the secondary outcome was cancer-specific mortality among twins with GORD and twins without GORD. Hazard ratios (HR) and 95% confidence intervals (CI) were analysed using parametric survival models, both in individual twin analyses and co-twin pair analyses, with adjustment for body mass index, smoking, education and comorbidity.

**Results:** Among 40,961 individual twins, 5,812 (14.2%) had GORD at baseline and 8,062 (19.7%) died during follow-up of up to 16 years. The risk of all-cause mortality (HR=1.00, 95% CI 0.94-1.07) and cancer-specific mortality (HR=0.99, 95% CI 0.89-1.10) were not increased in individual twins with GORD compared to individual twins without GORD. Similarly, there were no differences in mortality outcomes in within-pair analyses. The oesophageal adenocarcinoma-specific mortality rate was 0.45 (95% CI 0.32-0.66) per 1,000 person-years in individual twins with GORD and 0.22 (95% CI 0.18-0.27) per 1,000 person-years without GORD, rendering an adjusted HR of 2.01 (95% CI 1.35-2.98).


For peer review only

**Conclusions:** GORD did not increase all-cause or cancer-specific mortality when taking heredity and other confounders into account. The increased relative risk of mortality in oesophageal adenocarcinoma was low in absolute numbers.

**Keywords:** Survival; prognosis, heartburn; neoplasm; heredity; comorbidity

For peer review only



For peer review only - http://bmjopen.bmj.com/site/about/guidelines.xhtml

## Article Summary

### Strengths and limitations of this study

- The twin design which adjusts for heredity and shared familial confounders
- The prospective and nationwide population-based approach which counteracts recall and selection bias, as well as chance errors
- Valid and complete long-term follow-up using national registers
- Assessment of potential confounders
- No objective assessment of gastro-oesophageal reflux disease

For peer review only



For peer review only - http://bmjopen.bmj.com/site/about/guidelines.xhtml

## Introduction

Gastro-oesophageal reflux disease (GORD) is defined by troublesome heartburn and acid regurgitation occurring at least weekly or GORD-specific complications.[1] GORD affects between 10-30% of adults in the Western world and is one of the most common reasons for visits to gastroenterologists and general practitioners.[2, 3] Heredity, obesity, and tobacco smoking are the only established risk factors, while socioeconomic factors (mainly educational level) might also influence the risk of GORD.[4-7] Twin studies have shown that the heritability for GORD is 31-43%.[8, 9] Because GORD is associated with several conditions, e.g. cardiovascular disease, various gastrointestinal symptoms, anxiety, depression, sleep disorders,[10-13] reductions in health-related quality of life,[14, 15] and oesophageal and gastric cardia adenocarcinoma,[16] it has been hypothesised that GORD reduces life expectancy in general and increases mortality from cancer specifically. This is an important topic, not the least considering the high prevalence of GORD, and the consequences any influence on life expectancy would mean for healthcare and public health interventions. However, the research that has examined whether GORD increases the risk of mortality has been limited and provided conflicting results, some indicating a reduced survival and other not.[17-20] No previous study has taken the influence of all risk factors for GORD into account as confounders, particularly not heredity or shared familial exposures.

The present study aimed to clarify whether GORD influences the mortality for all causes, cancer in general, and oesophageal adenocarcinoma specifically by conducting a large and comprehensive twin study, controlling for genetic and familial influences, together with other potential confounders.

For peer review only

## Methods

Study design

This population-based twin study was based on data from the Swedish Twin Registry, during the study period 1998 through 2015. This Swedish Twin registry incorporates comprehensive data retrieved directly from twins combined with data collected from Swedish national health registries. The personal identity number, which is assigned to each Swedish inhabitant, enabled exact linkage of participants' data between the data sources.[21]

Ethical Approval and Patient Consent

The study was approved by the Regional Ethical Review Board in Stockholm reference number 2010/582-31/1). All twins gave a broad informed consent for data collection and research when participating. The study protocol conforms to the ethical guidelines of the 1975 Declaration of Helsinki as reflected in a priori approval by the institution's human research committee.

Patient and Public Involvement

Patients or the public were not involved in the design, or conduct, or reporting, or dissemination plans of our research.

Cohort

For peer review only



For peer review only - http://bmjopen.bmj.com/site/about/guidelines.xhtml

The study cohort was based on data from the Swedish Twin Registry, the largest and most comprehensive twin registry globally.[22, 23] It was established in the late 1950s and includes virtually all twins born in Sweden from 1886 onwards. During 1998-2002, the Screening Across the Lifespan Twin study (SALT) was performed with structured computer-assisted telephone interviews of twins born in 1958 or earlier and recorded in the Twin Registry, including assessment of GORD symptoms and risk factors for GORD.[22, 23] Data from the SALT interviews were used to define the study cohort and to assess information about GORD and the potential confounders heredity, body mass index (BMI), tobacco smoking, and education. Zygosity was assessed by a separate questionnaire sent to the twins. The twins were defined as monozygotic if both twins in a same-sexed pair reported they were "alike as two peas in a pod" and as dizygotic if they reported to be "not more alike than siblings". This simple method has been shown to be 99% accurate in of determining zygosity compared to DNA-testing.[22] The Swedish Twin Registry is regularly updated with information from other nationwide Swedish registries, i.e. the Cause of Death Registry, Cancer Registry and Patient Registry, which are briefly presented below.

*The Swedish Cause of Death Registry* provided data on all-cause and cancer-specific mortality. This registry includes date of death and causes of death for all Swedish residents since 1961, regardless of whether they died in Sweden or abroad. The information about date of death and cause of death is 100% and 99% complete, respectively.[24, 25]

*The Swedish Cancer Registry* had information about the histological type of oesophageal cancer (adenocarcinoma). This registry started in 1958 and includes standardized records of all newly diagnosed malignancies in Sweden, including date of diagnosis, tumour site, and



For peer review only - http://bmjopen.bmj.com/site/about/guidelines.xhtml

histological type. Histological type is registered in accordance with the World Health Organization's classification of histology (C24). The general completeness of the registry is 96% and it is 98% complete regarding recording of oesophageal adenocarcinoma, and for these patients, the histological verification is 100% complete.[26, 27]

*The Swedish Patient Registry* contained data on comorbidity. The registry contains date and International Classification of Diseases (ICD) versions 9 and 10 codes of diagnoses from all inpatient healthcare in Sweden from 1987 onwards and all specialist outpatient healthcare since 2001. This registry has a positive predictive value of any primary diagnosis close to 100%.[28] Diagnoses registered three years before and three years after the SALT interviews were included in the assessment of comorbidity. This restriction in time was done to counteract misclassification of comorbidity due to different lengths of follow-up among the participating twins.

<u>Exposure</u>

The twins were defined as being exposed to GORD if they reported in the SALT interview to have: 1) heartburn at least weekly, 2) regurgitation at least weekly, or 3) retrosternal pain at least weekly combined with antacid relief.[1]

<u>Outcomes</u>

The main outcome was all-cause mortality, which included any deaths, regardless of cause. A secondary outcome was overall cancer-specific mortality, which included deaths related to any cancer (ICD-7 140-199 or ICD-10 C00-C97), excluding non-melanoma skin cancer (ICD-7 191 or ICD-10 C44). The other secondary outcome was oesophageal adenocarcinoma-



For peer review only - http://bmjopen.bmj.com/site/about/guidelines.xhtml

specific mortality, defined as deaths related to oesophageal or gastroesophageal junctional adenocarcinoma (ICD-7 150 or 151.1 and C24 096 or ICD-10 C15 or C16.0 and C24 096).

## Confounders

Data on BMI, tobacco smoking, and education were retrieved from the SALT interviews. BMI was calculated as the weight (kilograms) divided by the square height (meters). Smoking status included consumption of cigarettes, cigars, and pipe. The level of education was assessed by the highest reported completed education qualification. Data on comorbidity were collected from the Swedish Patient Registry. The Royal College of Surgeons version of the Charlson comorbidity index was used to define and classify comborbidity.[29, 30]

## Statistical analyses

Mortality rates per 1,000 person-years were compared between individuals with and without GORD for all three mortality outcomes. Parametric survival models with Weibull distribution and sandwich estimator for the variance clustered by the twins' pair identity were used to calculate hazard ratios (HR) with 95% confidence intervals (CI). These models correct for within twin pair dependency and help to avoid underestimation of the variance. The baseline hazard was modelled with both a linear and a quadratic time term, to allow for more flexibility to the baseline function as the relation between the baseline hazard and time was quadratic. Proportionality of the hazards was verified in all analyses. Time at risk was defined from the date of the SALT interview (1998-2002), i.e., when GORD was assessed, until the date of death or the end of the study period (December 31, 2015).



For peer review only - http://bmjopen.bmj.com/site/about/guidelines.xhtml

The mortality among twins with GORD was compared with the mortality among twins without GORD in a stepwise series of analyses. First, external control analyses were performed using all individual twins, comparing individual twins with GORD to individual twins without GORD. Second, within-pair co-twin analyses of dizygotic twins discordant for GORD were performed. Third, within-pair co-twin analyses of monozygotic twins discordant for GORD were conducted. In the two latter analyses, only complete twin pairs were included. These three analysis steps were performed for each mortality outcome.

Stepwise adjustments for confounders were performed. First, a basic model adjusted for age (continuous) and sex. Second, the results were additionally adjusted for BMI (categorised into <25, 25-30, or >30), smoking (never, former, or current), and years of completed education (0-9.5 years, 9.5-12.5 years, or >12.5 years). Third, the results were further adjusted for comorbidity (Charlson comorbidity index score 0, 1, or ≥2),[29] which was done to assess whether comorbidity could explain any association between GORD and mortality.

In order to examine effect modification, analyses were stratified by age (≤60 or >60 years) and sex (except for the monozygotic twin analyses). In the monozygotic twin analysis of men aged 40-60 years, the HRs were estimated with exponential distribution and sandwich estimator for the variance, clustered by the twins' pair identity in order for the model to converge. This result should be similar to the model with Weibull distribution, which did not converge in this analysis.



For peer review only - http://bmjopen.bmj.com/site/about/guidelines.xhtml

A senior biostatistician (GS) conducted the data management and statistical analysis following a pre-defined study protocol. The statistical analyses were performed using Stata MP version 15, StataCorp LP, College Station, TX, USA.

For peer review only



For peer review only - http://bmjopen.bmj.com/site/about/guidelines.xhtml

## Results

Participants

Among 43,350 individual twins who participated in SALT, 40,961 (95.5%) answered the questions relevant for the present study and were thus included in the final analysis. A flowchart describing the study cohort is shown in Figure 1. Among the participating twins, 8,062 (19.7%) died during follow-up of up to 16 years, including 2,845 (6.9%) from any cancer and 127 (0.3%) from oesophageal adenocarcinoma. Characteristics of the included twins with and without GORD are shown in Table 1. The median age was 56 years in both groups. In all, 14.2% had GORD and GORD was similarly common in both sexes and in both dizygotic and monozygotic twins. Compared to twins without GORD, the twins with GORD were more often overweight or obese, tobacco smokers, less educated, and diagnosed with comorbidities (Table 1). The study included 2,501 dizygotic twin pairs discordant for GORD and 749 monozygotic twin pairs discordant for GORD.

Mortality from any cause

The all-cause mortality rate of all individual twins was 16.2 (95% CI 15.3-17.2) per 1,000 person-years in twins with GORD and also 16.2 (95% CI 15.8-16.7) per 1,000 person-years in twins without GORD (Table 2). In dizygotic twin pairs discordant for GORD, the all-cause mortality rates were 13.3 (95% CI 12.1-14.7) per 1,000 person-years in twins with GORD and 13.3 (95% CI 12.2-14.7) per 1,000 person-years for their co-twins without GORD. In monozygotic twin pairs discordant for GORD, the all-cause mortality rates were 12.0 (95% CI 10.0-14.4) per 1,000 person-years in twins with GORD and 12.2 (95% CI 10.2-14.6) per 1,000 person-years in their co-twins without GORD.



For peer review only - http://bmjopen.bmj.com/site/about/guidelines.xhtml

The fully adjusted HR of all-cause mortality was 1.00 (95% CI 0.94-1.07) comparing all individual twins with GORD to individual twins without GORD (Table 3). In the dizygotic twin analysis, the corresponding HR was 0.99 (95% CI 0.87-1.14). In the monozygotic twin analysis, the adjusted HR was 1.11 (95% CI 0.87-1.40). The analyses stratified by sex and age showed similar HRs without any association between GORD and all-cause mortality (Table 4).

<u>Mortality from any cancer</u>

The overall cancer-specific mortality rate of all individual twins was 5.8 (95% CI 5.3-6.4) per 1,000 person-years in those with GORD and 5.7 (95% CI 5.5-6.0) per 1,000 person-years in those without GORD (Table 2). The dizygotic twin analysis also showed similar cancer-specific mortality rates in twins with GORD (4.9 [95% CI 4.2-5.7] per 1,000 person-years) and their co-twin without (4.9 8 [95% CI 4.2-5.7] per 1,000 person-years). In the monozygotic twin analysis, the corresponding rates were 5.2 (95% CI 3.9-6.9) per 1,000 person-years in twins with GORD and 4.6 (95% CI 3.5-6.3) per 1,000 person-years in their co-twins with no GORD.

The fully adjusted HR of overall cancer-specific mortality was 0.99 (95% CI 0.89-1.10) comparing all individual twins with GORD to individual twins without GORD (Table 3). The corresponding HRs in dizygotic twins and monozygotic twins were 0.99 (95% CI 0.78-1.24) and 1.28 (95% CI 0.87-1.87), respectively. The analyses stratified by sex and age showed similar HRs and no association between GORD and overall cancer-specific mortality (Table 4).



For peer review only - http://bmjopen.bmj.com/site/about/guidelines.xhtml

Mortality from oesophageal adenocarcinoma

The oesophageal adenocarcinoma-specific mortality rate was 0.45 (95% CI 0.32-0.66) per 1,000 person-years in all individual twins with GORD, compared to 0.22 (95% CI 0.18-0.27) per 1,000 person-years in twins without GORD (Table 2). In dizygotic twins, this rate was 0.39 (95% CI 0.23-0.74) per 1,000 person-years in the twins with GORD and 0.26 (95% CI 0.13-0.58) per 1,000 person-years in the twins without GORD. The mortality rate was 0.32 (95% CI 0.10-1.58) per 1,000 person-years in the monozygotic twins with GORD, while there was no oesophageal adenocarcinoma-specific mortality in the monozygotic twins without GORD.

The fully adjusted HR was 2.01 (95% CI 1.35-2.98) for oesophageal adenocarcinoma-specific mortality comparing all individual twins with GORD to those without GORD (Table 3). In dizygotic twins, the corresponding HR was 1.44 (95% CI 0.60-3.45), while the statistical power was insufficient for monozygotic twin analysis. The HR was 3.71 (95% CI 1.90-7.28) in men aged 40-60 years, and 1.60 (95% CI 0.77-3.32) in men aged >60 years (Table 4). The stratified dizygotic twin analyses had low statistical power, but the fully adjusted HR for oesophageal adenocarcinoma-specific mortality was 2.07 (95% CI 0.53-8.08) among men aged 40-60 years and 0.82 (95% CI 0.15-4.61) among men aged >60 years.

For peer review only

## Discussion

This large-scale twin study found no increased all-cause or cancer-specific mortality in twins with GORD compared to twins without GORD. The risk of mortality in oesophageal adenocarcinoma was higher in twins with GORD than in twins without GORD, but the absolute risk was still low.

Among methodological strengths is the twin design, which enabled the first study on the topic with adjustment for heredity and shared familial confounders. The prospective and nationwide population-based approach counteracted recall and selection bias, as well as chance errors. The high-quality and complete data reduced misclassification and enabled long and complete follow-up of all participants. The assessment of mortality was valid and complete. The definition of GORD was the evidence-based Montreal consensus, which remains the definition of choice for research purposes.[1] The prevalence of GORD in this study coincides well with the prevalence reported in similar Western populations,[2] indicating validity of the definition of GORD. The assessment of potential confounders through the structured SALT interviews (BMI, tobacco smoking, and education) and the Patient Registry (comorbidity) allowed for adjustment of all risk factors for GORD and mortality, i.e. all plausible confounders. The rate of missing values for the variables included in the study was low, and all analyses were complete case analyses. The large sample size allowed for age and sex stratified analyses to assess effect modification with age and sex.

There are also limitations. Some level of misclassification of GORD could not be avoided. Residual or unmeasured confounding cannot be ruled out in this observational study. The study lacks information on medical and surgical treatment of GORD, so any change in



For peer review only - http://bmjopen.bmj.com/site/about/guidelines.xhtml

mortality related to treatment could not be assessed. The dizygotic and monozygotic co-twin analyses had limited statistical power, although the results generally supported the overall findings.

The results of the present study showing no increased all-cause mortality in individuals with GORD corroborates the findings of our recent cohort study from Norway,[20] a cohort study from the United States,[18] and a cohort study from Iran.[19] However, three cohort studies from the United Kingdom showed a 1.16- to 1.6-fold increase in mortality in people with GORD compared with the background population, the majority of deaths being due to cardiac disease.[17] The increased mortality found in some studies could be due to prevalent cancers provoking GORD symptoms. No earlier study has heredity as a confounder, although heredity is a strong risk factor for GORD.[8]

GORD is common in Western populations, with 10-30% prevalence in adults [2, 3]. The present study implies that individuals with GORD do not need to worry about any increased risk of dying. The increased risk of death from oesophageal adenocarcinoma should not be overemphasized because the absolute risk is still low even in the presence of GORD. However, if the incidence of oesophageal adenocarcinoma continues to increase strongly without any improvements in the survival, the influence of mortality from this tumour could increase.

In conclusion, this nationwide Swedish population-based cohort study in twins with long and complete follow-up and adjustment for confounders indicates that GORD does not increase the risk of all-cause or cancer-specific mortality. Despite the increased relative risk of



For peer review only - http://bmjopen.bmj.com/site/about/guidelines.xhtml

mortality from oesophageal adenocarcinoma in individuals with GORD, the absolute risk is still low.

For peer review only



For peer review only - http://bmjopen.bmj.com/site/about/guidelines.xhtml

**Acknowledgments**

We are grateful to the Swedish Twin Registry for access to data. This Registry is managed by Karolinska Institutet and receives funding from the Swedish Research Council (grant 2017-00641).

**Disclosure of interest**

The authors report no conflict of interest.

**Funding**

This work was supported by the Swedish Research Council (SIMSAM) under grant number 2017-00641; Swedish Society of Medicine; and the United European Gastroenterology (UEG Research Prize to JL). The funders did not have any role in the study design, in the collection, analysis, or interpretation of data.

**Data availability statement**

The data set used in this paper is available through application to The Swedish Twin Registry (https://ki.se/en/research/swedish-twin-registry-for-researchers)

**Author statement**

(i) Guarantor of the article: Eivind Ness-Jensen; (ii) Specific author contributions: **ENJ:** study concept and design; acquisition of data; analysis and interpretation of data; drafting of the manuscript; critical revision of the manuscript for important intellectual content; obtained funding. **GS:** study concept and design; analysis and interpretation of data; critical revision of the manuscript for important intellectual content; statistical analysis. **EGV:** study concept



For peer review only - http://bmjopen.bmj.com/site/about/guidelines.xhtml

For peer review only

and design; acquisition of data; analysis and interpretation of data; critical revision of the manuscript for important intellectual content. **AL:** study concept and design; acquisition of data; analysis and interpretation of data; critical revision of the manuscript for important intellectual content. **NP:** study concept and design; acquisition of data; analysis and interpretation of data; critical revision of the manuscript for important intellectual content. **JL:** study concept and design; analysis and interpretation of data; critical revision of the manuscript for important intellectual content; obtained funding; (iii) All authors approved the final version of the manuscript.

For peer review only

## References


1. Vakil N, van Zanten SV, Kahrilas P, et al. The Montreal definition and classification of gastroesophageal reflux disease: a global evidence-based consensus. Am J Gastroenterol 2006;101:1900-20; quiz 1943.
2. El-Serag HB, Sweet S, Winchester CC, et al. Update on the epidemiology of gastro-oesophageal reflux disease: a systematic review. Gut 2014;63:871-80.
3. Ness-Jensen E, Lindam A, Lagergren J, et al. Changes in prevalence, incidence and spontaneous loss of gastro-oesophageal reflux symptoms: a prospective population-based cohort study, the HUNT study. Gut 2012;61:1390-7.
4. Dent J, El-Serag HB, Wallander MA, et al. Epidemiology of gastro-oesophageal reflux disease: a systematic review. Gut 2005;54:710-7.
5. Nilsson M, Johnsen R, Ye W, et al. Lifestyle related risk factors in the aetiology of gastro-oesophageal reflux. Gut 2004;53:1730-5.
6. Jansson C, Nordenstedt H, Johansson S, et al. Relation between gastroesophageal reflux symptoms and socioeconomic factors: a population-based study (the HUNT Study). Clin Gastroenterol Hepatol 2007;5:1029-34.
7. Hallan A, Bomme M, Hveem K, et al. Risk factors on the development of new-onset gastroesophageal reflux symptoms. A population-based prospective cohort study: the HUNT study. Am J Gastroenterol 2015;110:393-400; quiz 401.
8. Cameron AJ, Lagergren J, Henriksson C, et al. Gastroesophageal reflux disease in monozygotic and dizygotic twins. Gastroenterology 2002;122:55-9.
9. Mohammed I, Cherkas LF, Riley SA, et al. Genetic influences in gastro-oesophageal reflux disease: a twin study. Gut 2003;52:1085-9.
10. Jansson C, Nordenstedt H, Wallander MA, et al. Severe gastro-oesophageal reflux symptoms in relation to anxiety, depression and coping in a population-based study. Aliment Pharmacol Ther 2007;26:683-91.
11. Jansson C, Nordenstedt H, Wallander MA, et al. Severe symptoms of gastro-oesophageal reflux disease are associated with cardiovascular disease and other gastrointestinal symptoms, but not diabetes: a population-based study. Aliment Pharmacol Ther 2008;27:58-65.
12. Lindam A, Jansson C, Nordenstedt H, et al. A population-based study of gastroesophageal reflux disease and sleep problems in elderly twins. PLoS One 2012;7:e48602.
13. Lindam A, Ness-Jensen E, Jansson C, et al. Gastroesophageal Reflux and Sleep Disturbances: A Bidirectional Association in a Population-Based Cohort Study, The HUNT Study. Sleep 2016;39:1421-7.
14. Wiklund I, Carlsson J, Vakil N. Gastroesophageal reflux symptoms and well-being in a random sample of the general population of a Swedish community. Am J Gastroenterol 2006;101:18-28.
15. Ronkainen J, Aro P, Storskrubb T, et al. Gastro-oesophageal reflux symptoms and health-related quality of life in the adult general population--the Kalixanda study. Aliment Pharmacol Ther 2006;23:1725-33.
16. Lagergren J, Bergstrom R, Lindgren A, et al. Symptomatic gastroesophageal reflux as a risk factor for esophageal adenocarcinoma. N Engl J Med 1999;340:825-31.
17. Becher A, El-Serag HB. Mortality associated with gastroesophageal reflux disease and its non-malignant complications: a systematic review. Scand J Gastroenterol 2008;43:645-53.
18. Talley NJ, Locke GR, 3rd, McNally M, et al. Impact of gastroesophageal reflux on survival in the community. Am J Gastroenterol 2008;103:12-9.
19. Islami F, Pourshams A, Nasseri-Moghaddam S, et al. Gastroesophageal Reflux Disease and overall and Cause-specific Mortality: A Prospective Study of 50000 Individuals. Middle East J Dig Dis 2014;6:65-80.




For peer review only - http://bmjopen.bmj.com/site/about/guidelines.xhtml

20. Ness-Jensen E, Gottlieb-Vedi E, Wahlin K, et al. All-cause and cancer-specific mortality in GORD in a population-based cohort study (the HUNT study). Gut 2018;67:209-215.
21. Ludvigsson JF, Otterblad-Olausson P, Pettersson BU, et al. The Swedish personal identity number: possibilities and pitfalls in healthcare and medical research. Eur J Epidemiol 2009;24:659-67.
22. Lichtenstein P, De Faire U, Floderus B, et al. The Swedish Twin Registry: a unique resource for clinical, epidemiological and genetic studies. J Intern Med 2002;252:184-205.
23. Lichtenstein P, Sullivan PF, Cnattingius S, et al. The Swedish Twin Registry in the third millennium: an update. Twin Res Hum Genet 2006;9:875-82.
24. Johansson LA, Westerling R. Comparing Swedish hospital discharge records with death certificates: implications for mortality statistics. Int J Epidemiol 2000;29:495-502.
25. Brooke HL, Talback M, Hornblad J, et al. The Swedish cause of death register. Eur J Epidemiol 2017;32:765-773.
26. Barlow L, Westergren K, Holmberg L, et al. The completeness of the Swedish Cancer Register: a sample survey for year 1998. Acta Oncol 2009;48:27-33.
27. Lindblad M, Ye W, Lindgren A, et al. Disparities in the classification of esophageal and cardia adenocarcinomas and their influence on reported incidence rates. Ann Surg 2006;243:479-85.
28. Ludvigsson JF, Andersson E, Ekbom A, et al. External review and validation of the Swedish national inpatient register. BMC Public Health 2011;11:450.
29. Armitage JN, van der Meulen JH, Royal College of Surgeons Co-morbidity Consensus G. Identifying co-morbidity in surgical patients using administrative data with the Royal College of Surgeons Charlson Score. Br J Surg 2010;97:772-81.
30. Brusselaers N, Lagergren J. The Charlson Comorbidity Index in Registry-based Research. Methods Inf Med 2017;56:401-406.

For peer review only

**Table 1.** Baseline characteristics of twins with and without gastro-oesophageal reflux disease (GORD)

| | GORD | | No GORD | |
|---|---|---|---|---|
| | Number (%) | | Number (%) | |
| Total | 5,812 | (14.2) | 35,149 | (85.8) |
| Age, years* | 56 | (41-95) | 56 | (41-99) |
| Sex | | | | |
| Men | 2,673 | (46.0) | 16,683 | (47.5) |
| Women | 3,139 | (54.0) | 18,466 | (52.5) |
| Zygosity | | | | |
| Monozygotic | 1,444 | (24-8) | 8,860 | (25.2) |
| Dizygotic | 4,368 | (75.2) | 26,289 | (75.8) |
| BMI | | | | |
| <25 | 2,568 | (44.2) | 19,577 | (55.7) |
| 25-30 | 2,535 | (43.6) | 12,909 | (36.7) |
| >30 | 709 | (12.2) | 2,663 | (7.6) |
| Tobacco smoking-status | | | | |
| Never | 1,236 | (21.3) | 8,985 | (25.6) |
| Former | 3,330 | (57.3) | 19,104 | (54.4) |
| Current | 1,246 | (21.4) | 7,060 | (20.1) |
| Education, years | | | | |
| 0-9.5 | 3,019 | (51.9) | 16,405 | (46.7) |
| 9.5-12.5 | 1,625 | (28.0) | 9,895 | (28.2) |
| >12.5 | 1,168 | (20.1) | 1,168 | (25.2) |
| Charlson co-morbidity index | | | | |
| 0 | 5,190 | (89.3) | 31,820 | (90.5) |
| 1 | 524 | (9.0) | 2,883 | (8.2) |
| >1 | 98 | (1.7) | 446 | (1.3) |

*Median (range)

For peer review only

**Table 2.** Number of deaths and mortality rates for all-cause, cancer-specific, and oesophageal adenocarcinoma (OAC)-specific mortality in twins with and without gastro-oesophageal reflux disease (GORD)

| | Alive (number) | | Deaths (number) | | Mortality rates per 1,000 person-years (95% CI) | |
|---|---|---|---|---|---|---|
| **Outcome** | **GORD** | **No GORD** | **GORD** | **No GORD** | **GORD** | **No GORD** |
| All-cause mortality | | | | | | |
| All twins | 6,922 | 28,227 | 1,140 | 4,672 | 16.2 (15.3-17.2) | 16.2 (15.8-16.7) |
| Dizygotic[a] | 2,091 | 2,090 | 410 | 411 | 13.3 (12.1-14.7) | 13.3 (12.2-14.7) |
| Monozygotic[a] | 638 | 636 | 111 | 113 | 12.0 (10.0-14.4) | 12.2 (10.2-14.6) |
| Overall cancer-specific mortality | | | | | | |
| All twins | 5,404 | 32,707 | 408 | 2437 | 5.8 (5.3-6.4) | 5.7 (5.5-6.0) |
| Dizygotic[a] | 2,351 | 2,351 | 150 | 150 | 4.9 (4.2-5.7) | 4.9 (4.2-5.7) |
| Monozygotic[a] | 701 | 706 | 48 | 43 | 5.2 (3.9-6.9) | 4.6 (3.5-6.3) |
| OAC-specific mortality | | | | | | |
| All twins | 5,780 | 35,049 | 32 | 95 | 0.45 (0.32-0.66) | 0.22 (0.18-0.27) |
| Dizygotic[a] | 2,489 | 2,493 | 12 | 8 | 0.39 (0.23-0.74) | 0.26 (0.13-0.58) |
| Monozygotic[a] | 746 | 749 | 3 | 0 | 0.32 (0.10-1.58) | - |

[a]Discordant for gastro-oesophageal reflux disease

For peer review only



For peer review only - http://bmjopen.bmj.com/site/about/guidelines.xhtml

**Table 3.** Hazard ratio (HR) with 95% confidence interval (CI) for all-cause, cancer-specific, and oesophageal adenocarcinoma (OAC)-specific-specific mortality in twins with and without gastro-oesophageal reflux disease

| | Model 1[a] | | | Model 2[b] | | | Model 3[c] | | |
|---|---|---|---|---|---|---|---|---|---|
| **Outcome** | **HR** | **(95% CI)** | | **HR** | **(95% CI)** | | **HR** | **(95% CI)** | |
| All-cause mortality | | | | | | | | | |
| All twins | 1.03 | (0.97 | 1.10) | 1.03 | (0.96 | 1.09) | 1.00 | (0.94 | 1.07) |
| Dizygotic[d] | 0.99 | (0.87 | 1.13) | 1.04 | (0.91 | 1.18) | 0.99 | (0.87 | 1.14) |
| Monozygotic[d] | 0.99 | (0.79 | 1.24) | 1.05 | (0.84 | 1.32) | 1.11 | (0.87 | 1.40) |
| Overall cancer-specific mortality | | | | | | | | | |
| All twins | 1.04 | (0.93 | 1.15) | 1.02 | (0.92 | 1.14) | 0.99 | (0.89 | 1.10) |
| Dizygotic[d] | 1.00 | (0.80 | 1.25) | 1.04 | (0.83 | 1.30) | 0.99 | (0.78 | 1.24) |
| Monozygotic[d] | 1.13 | (0.78 | 1.62) | 1.21 | (0.84 | 1.75) | 1.28 | (0.87 | 1.87) |
| OAC-specific mortality | | | | | | | | | |
| All twins | 2.09 | (1.40 | 3.13) | 2.11 | (1.41 | 3.15) | 2.01 | (1.35 | 2.98) |
| Dizygotic[d] | 1.50 | (0.61 | 3.68) | 1.62 | (0.70 | 3.78) | 1.44 | (0.60 | 3.45) |
| Monozygotic[d] | - | - | - | - | - | - | - | - | - |

[a]Adjusted for age and sex

[b]Adjusted for age, sex, BMI, tobacco smoking status, and education

[c]Adjusted for age, sex, BMI, tobacco smoking status, education, and Charlson comorbidity index

[d]Discordant for gastro-oesophageal reflux disease

For peer review only

**Table 4.** Hazard ratio[a] (HR) with 95% confidence interval (CI) for all-cause, cancer-specific, and oesophageal adenocarcinoma (OAC)-specific mortality in twins with and without gastro-oesophageal reflux disease (GORD)

| | Age 40-60 years | | | | | Age >60 years | | | | |
|---|---|---|---|---|---|---|---|---|---|---|
| | Number of deaths | | | | | Number of deaths | | | | |
| **Outcome** | **GORD** | **No GORD** | **HR** | **(95% CI)** | | **GORD** | **No GORD** | **HR** | **(95% CI)** | |
| **Men** | | | | | | | | | | |
| All-cause mortality | | | | | | | | | | |
| All twins | 140 | 766 | 0.97 | (0.80 | 1.17) | 437 | 2,885 | 0.96 | (0.87 | 1.06) |
| Dizygotic twins[a] | 58 | 54 | 0.96 | (0.66 | 1.39) | 152 | 164 | 0.91 | (0.73 | 1.13) |
| Monozygotic twins[a] | 16 | 16 | 1.09 | (0.57 | 2.15) | 38 | 32 | 1.40 | (0.92 | 2.13) |
| Overall cancer-specific mortality | | | | | | | | | | |
| All twins | 61 | 327 | 0.97 | (0.73 | 1.29) | 151 | 971 | 0.97 | (0.81 | 1.15) |
| Dizygotic twins[a] | 27 | 24 | 1.06 | (0.60 | 1.87) | 51 | 57 | 0.80 | (0.54 | 1.18) |
| Monozygotic twins[a] | 3 | 5 | 0.65 | (0.12 | 3.38)[e] | 20 | 13 | 1.80 | (0.96 | 3.38) |
| OAC-specific mortality | | | | | | | | | | |
| All twins | 14 | 22 | 3.71 | (1.90 | 7.28) | 9 | 36 | 1.60 | (0.77 | 3.32) |
| Dizygotic twins[a] | 6 | 1 | 2.07 | (0.53 | 8.08) | 2 | 3 | 0.82 | (0.15 | 4.61) |
| Monozygotic twins[a] | 1 | 0 | - | - | - | 2 | 0 | - | - | - |
| **Women** | | | | | | | | | | |
| All-cause mortality | | | | | | | | | | |
| All twins | 133 | 676 | 1.03 | (0.85 | 1.26) | 430 | 2,595 | 1.00 | (0.90 | 1.11) |
| Dizygotic twins[a] | 52 | 56 | 1.03 | (0.70 | 1.51) | 148 | 137 | 1.10 | (0.87 | 1.40) |
| Monozygotic twins[a] | 15 | 20 | 0.75 | (0.34 | 1.67) | 42 | 45 | 1.11 | (0.78 | 1.57) |
| Overall cancer-specific mortality | | | | | | | | | | |
| All twins | 65 | 427 | 0.80 | (0.61 | 1.05) | 131 | 712 | 1.10 | (0.91 | 1.33) |
| Dizygotic twins[a] | 23 | 28 | 0.93 | (0.52 | 1.65) | 49 | 41 | 1.30 | (0.84 | 2.03) |
| Monozygotic twins[a] | 11 | 11 | 1.07 | (0.41 | 2.77) | 14 | 14 | 1.06 | (0.52 | 2.15) |
| OAC-specific mortality | | | | | | | | | | |
| All twins | 1 | 9 | 0.51 | (0.06 | 4.09) | 8 | 28 | 1.81 | (0.83 | 3.94) |
| Dizygotic twins[a] | 0 | 0 | - | - | - | 3 | 2 | 1.39 | (0.30 | 6.43) |
| Monozygotic twins[a] | 0 | 0 | - | - | - | 0 | 0 | - | - | - |

[a]Discordant for gastro-oesophageal reflux disease



For peer review only - http://bmjopen.bmj.com/site/about/guidelines.xhtml

**Figure 1.** Study population, sample, and vital status in twins with and without gastro-oesophageal reflux disease (GORD)

*SALT: Screening Across the Lifespan Twin cohort

§OAC: Oesophageal adenocarcinom

For peer review only



For peer review only - http://bmjopen.bmj.com/site/about/guidelines.xhtml

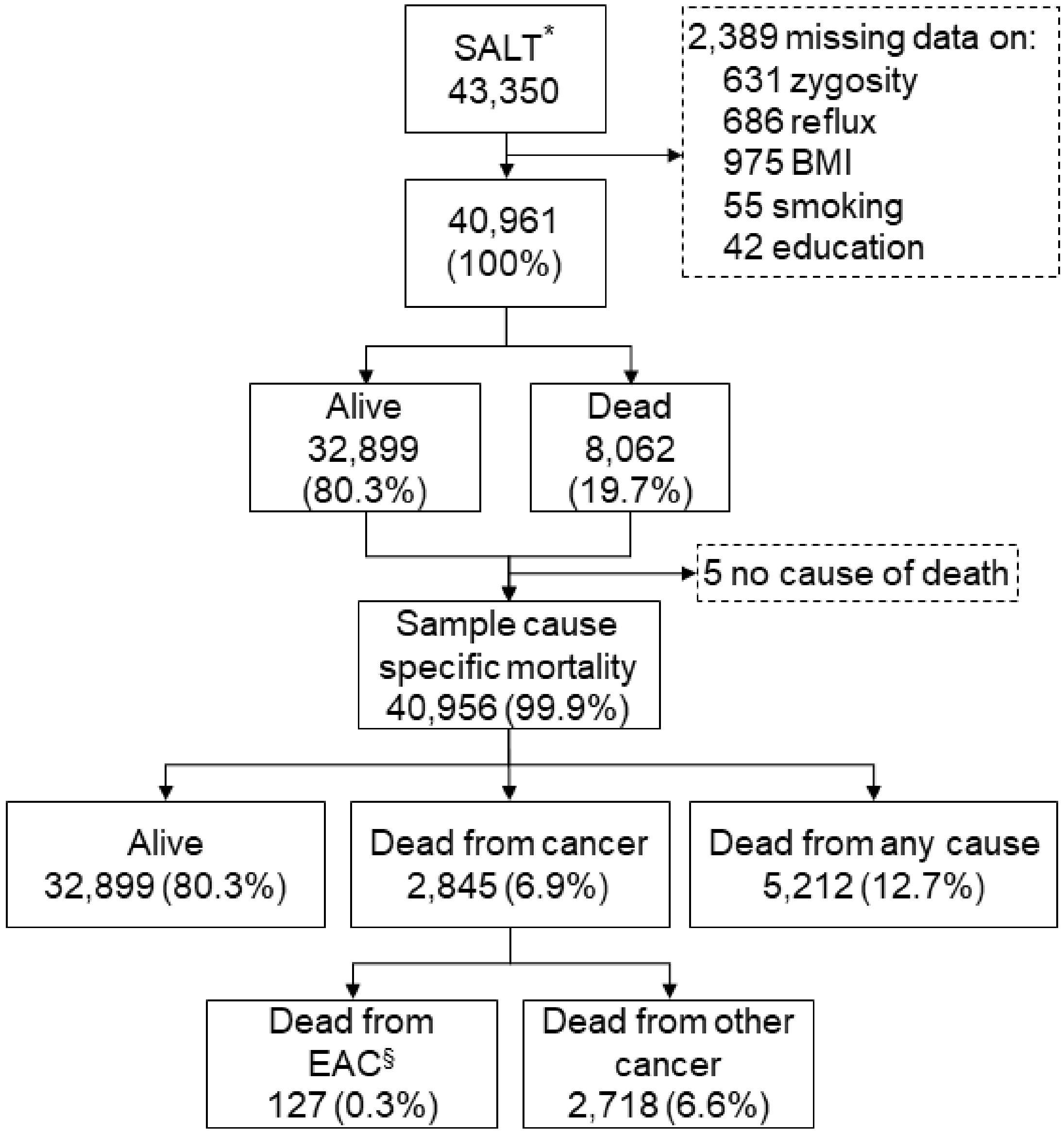
SALT*
43,350
2,389 missing data on:
631 zygosity
686 reflux
975 BMI
55 smoking
42 education
40,961
(100%)
Alive
32,899
(80.3%)
Dead
8,062
(19.7%)
5 no cause of death
Sample cause
specific mortality
40,956 (99.9%)
Alive
32,899 (80.3%)
Dead from cancer
2,845 (6.9%)
Dead from any cause
5,212 (12.7%)
Dead from
EAC§
127 (0.3%)
Dead from other
cancer
2,718 (6.6%)

STROBE Statement—Checklist of items that should be included in reports of ***cohort studies***

| | Item No | Recommendation |
|---|---|---|
| **Title and abstract** | 1 | (*a*) Indicate the study's design with a commonly used term in the title or the abstract **Page 1** |
| | | (*b*) Provide in the abstract an informative and balanced summary of what was done and what was found **Page 2** |
| **Introduction** | | |
| Background/rationale | 2 | Explain the scientific background and rationale for the investigation being reported **Page 5** |
| Objectives | 3 | State specific objectives, including any prespecified hypotheses **Page 5** |
| **Methods** | | |
| Study design | 4 | Present key elements of study design early in the paper **Page 6** |
| Setting | 5 | Describe the setting, locations, and relevant dates, including periods of recruitment, exposure, follow-up, and data collection **Page 6** |
| Participants | 6 | (*a*) Give the eligibility criteria, and the sources and methods of selection of participants. Describe methods of follow-up **Pages 6 to 7** |
| | | (*b*) For matched studies, give matching criteria and number of exposed and unexposed **NA** |
| Variables | 7 | Clearly define all outcomes, exposures, predictors, potential confounders, and effect modifiers. Give diagnostic criteria, if applicable **Pages 6 to 8** |
| Data sources/ measurement | 8* | For each variable of interest, give sources of data and details of methods of assessment (measurement). Describe comparability of assessment methods if there is more than one group **Pages 6 to 8** |
| Bias | 9 | Describe any efforts to address potential sources of bias **Pages 9 to 10** |
| Study size | 10 | Explain how the study size was arrived at **Page 11 and Figure** |
| Quantitative variables | 11 | Explain how quantitative variables were handled in the analyses. If applicable, describe which groupings were chosen and why **Page 9** |
| Statistical methods | 12 | (*a*) Describe all statistical methods, including those used to control for confounding **Pages 9 to 10** |
| | | (*b*) Describe any methods used to examine subgroups and interactions **Pages 9 to 10** |
| | | (*c*) Explain how missing data were addressed **Page 14 and Figure** |
| | | (*d*) If applicable, explain how loss to follow-up was addressed **NA** |
| | | (*e*) Describe any sensitivity analyses |

For peer review only

| | | |
|---|---|---|
| | | **NA** |
| **Results** | | |
| Participants | 13* | (a) Report numbers of individuals at each stage of study—eg numbers potentially eligible, examined for eligibility, confirmed eligible, included in the study, completing follow-up, and analysed<br>**Page 11 and Figure** |
| | | (b) Give reasons for non-participation at each stage<br>**Page 11 and Figure** |
| | | (c) Consider use of a flow diagram<br>**Figure** |
| Descriptive data | 14* | (a) Give characteristics of study participants (eg demographic, clinical, social) and information on exposures and potential confounders<br>**Page 11 and Table 1** |
| | | (b) Indicate number of participants with missing data for each variable of interest<br>**Figure** |
| | | (c) Summarise follow-up time (eg, average and total amount)<br>**Page 11** |
| Outcome data | 15* | Report numbers of outcome events or summary measures over time<br>**Tables 2 and 4** |
| Main results | 16 | (*a*) Give unadjusted estimates and, if applicable, confounder-adjusted estimates and their precision (eg, 95% confidence interval). Make clear which confounders were adjusted for and why they were included<br>**Pages 11 to 13 and Tables 2 to 4** |
| | | (*b*) Report category boundaries when continuous variables were categorized<br>**Page 9** |
| | | (*c*) If relevant, consider translating estimates of relative risk into absolute risk for a meaningful time period<br>**Pages 11 to 13 and Table 2** |
| Other analyses | 17 | Report other analyses done—eg analyses of subgroups and interactions, and sensitivity analyses<br>**Pages 11 to 13 and Table 4** |
| **Discussion** | | |
| Key results | 18 | Summarise key results with reference to study objectives<br>**Page 14** |
| Limitations | 19 | Discuss limitations of the study, taking into account sources of potential bias or imprecision. Discuss both direction and magnitude of any potential bias<br>**Page 14** |
| Interpretation | 20 | Give a cautious overall interpretation of results considering objectives, limitations, multiplicity of analyses, results from similar studies, and other relevant evidence<br>**Pages 14 to 15** |
| Generalisability | 21 | Discuss the generalisability (external validity) of the study results<br>**Page 15** |
| **Other information** | | |
| Funding | 22 | Give the source of funding and the role of the funders for the present study and, if applicable, for the original study on which the present article is based<br>**Page 16** |

*Give information separately for exposed and unexposed groups.



For peer review only - http://bmjopen.bmj.com/site/about/guidelines.xhtml

For peer review only

**Note:** An Explanation and Elaboration article discusses each checklist item and gives methodological background and published examples of transparent reporting. The STROBE checklist is best used in conjunction with this article (freely available on the Web sites of PLoS Medicine at http://www.plosmedicine.org/, Annals of Internal Medicine at http://www.annals.org/, and Epidemiology at http://www.epidem.com/). Information on the STROBE Initiative is available at http://www.strobe-statement.org.

For peer review only



For peer review only - http://bmjopen.bmj.com/site/about/guidelines.xhtml
